# A New Screen for Tuberculosis Drug Candidates Utilizing a Luciferase-Expressing Recombinant *Mycobacterium bovis* Bacillus Calmette-Guéren

**DOI:** 10.1371/journal.pone.0141658

**Published:** 2015-11-16

**Authors:** Yuriko Ozeki, Masayuki Igarashi, Matsumi Doe, Aki Tamaru, Naoko Kinoshita, Yoshitoshi Ogura, Tomotada Iwamoto, Ryuichi Sawa, Maya Umekita, Shymaa Enany, Yukiko Nishiuchi, Mayuko Osada-Oka, Tetsuya Hayashi, Mamiko Niki, Yoshitaka Tateishi, Masaki Hatano, Sohkichi Matsumoto

**Affiliations:** 1 Department of Bacteriology, Graduate School of Medical and Dental Sciences, Niigata University, Niigata, Japan; 2 Department of Microbiology, Institute of Microbial Chemistry, Microbial Chemistry Research Foundation, Tokyo, Japan; 3 Graduate School of Sciences, Osaka City University, Osaka, Japan; 4 Bacteriology Division, Osaka Prefectural Institute of Public Health, Osaka, Japan; 5 Division of Bioenvironmental Science, Frontier Science Research Center, University of Miyazaki, Miyazaki, Japan; 6 Department of Microbiology, Kobe Institute of Health, Kobe, Japan; 7 Department of Microbiology and Immunology, Faculty of Pharmacy, Suez Canal University, Ismailia, Egypt; 8 Toneyama Institute for Tuberculosis Research, Osaka City University Medical School, Osaka, Japan; 9 Food Hygiene and Environmental Health Division of Applied Life Science, Graduate School of Life and Environmental Sciences, Kyoto Prefectural University, Kyoto, Japan; 10 Department of Bacteriology, Osaka City University Graduate School of Medicine, Osaka, Japan; Public Health England, UNITED KINGDOM

## Abstract

Tuberculosis (TB) is a serious infectious disease caused by a bacterial pathogen. Mortality from tuberculosis was estimated at 1.5 million deaths worldwide in 2013. Development of new TB drugs is needed to not only to shorten the medication period but also to treat multi-drug resistant and extensively drug-resistant TB. *Mycobacterium tuberculosis* (Mtb) grows slowly and only multiplies once or twice per day. Therefore, conventional drug screening takes more than 3 weeks. Additionally, a biosafety level-3 (BSL-3) facility is required. Thus, we developed a new screening method to identify TB drug candidates by utilizing luciferase-expressing recombinant *Mycobacterium bovis* bacillus Calmette-Guéren (rBCG). Using this method, we identified several candidates in 4 days in a non-BSL-3 facility. We screened 10,080 individual crude extracts derived from *Actinomyces* and *Streptomyces* and identified 137 extracts which possessed suppressive activity to the luciferase of rBCG. Among them, 41 compounds inhibited the growth of both Mtb H37Rv and the extensively drug-resistant Mtb (XDR-Mtb) strains. We purified the active substance of the 1904–1 extract, which possessed strong activity toward rBCG, Mtb H37Rv, and XDR-Mtb but was harmless to the host eukaryotic cells. The MIC of this substance was 0.13 μg/ml, 0.5 μg/ml, and 2.0–7.5 μg/ml against rBCG, H37Rv, and 2 XDR-strains, respectively. Its efficacy was specific to acid-fast bacterium except for the *Mycobacterium avium intracellular* complex. Mass spectrometry and nuclear magnetic resonance analyses revealed that the active substance of 1904–1 was cyclomarin A. To confirm the mode of action of the 1904-1-derived compound, resistant BCG clones were used. Whole genome DNA sequence analysis showed that these clones contained a mutation in the *clpc* gene which encodes caseinolytic protein, an essential component of an ATP-dependent proteinase, and the likely target of the active substance of 1904–1. Our method provides a rapid and convenient screen to identify an anti-mycobacterial drug.

## Introduction

Tuberculosis (TB) is a disease caused by *Mycobacterium tuberculosis* (Mtb) and remains a health-threatening serious problem in the world. In 2013, 9 million people developed TB and nearly 1.5 million died as a result of the infection [1]. Chemotherapies have been established against TB since the discovery of streptomycin (SM). The current effective short-course chemotherapy (DOTS) takes at least 6 months for the treatment of active TB. For latent tuberculosis infection (LTBI), a course of 3 to 9 months of medications is currently the recommended treatment regimen. As the treatment time increases, more patients dropout from the therapy, resulting in the generation of additional drug resistant strains.

Emerging multi-drug resistant Mtb (MDR-Mtb), especially those strains which are resistant to isoniazid (INH) and rifampicin (RFP), have complicated TB therapy. More recently, extensively drug-resistant Mtb (XDR-Mtb), which is resistant to all fluoroquinolones and at least one of the three injectable second-line drugs in addition to INH and RFP, has been reported. Mtb is considered a serious threat to human health [2, 3]. Thus, establishment of new TB drugs that can be administrated over a short term and capable of controlling the emergence of MDR- and XDR-TB are critically urgent, even after the recent approval of two new TB drugs for clinical use [4–6].

Several characteristics of Mtb are considered barriers for TB drug development including its slow growing rate, high virulence, and its contagiousness. Moreover, Mtb must be manipulated in a biosafety laboratory level 3 (BSL-3), which requires a specialized facility and a high cost to maintain.

*Mycobacterium bovis* Bacillus Calmette-Guérin (BCG) is an attenuated strain of *Mycobacterium bovis*, genetically related to the Mtb complex. It is safe and has been widely used as a TB vaccine for humans. Although BCG is avirulent, it possesses many biological characteristics of Mtb. BCG displays the same responses to drugs as Mtb because they share the essential genes for survival. All front and second line TB drugs developed to date are actually effective toward BCG. Since the development of the host-vector system of mycobacteria [7], recombinant BCG (rBCG) has been used for the development of more effective TB vaccines [8, 9] and new preventive or therapeutic agents against other serious diseases [10–15].

Firefly luciferase [16] has been widely used as a reporter to assess gene expression in various organisms including both eukaryotes and prokaryotes. This enzyme hydrolyses luciferin in an ATP-dependent manner and releases the photons which can be measured by a sensitive light-detection system. Because ATP is abundant in living cells but rapidly hydrolyzed in non-viable cells, the amount of photons released by the enzymatic activity of intracellular luciferase can be correlated with the viability of the cells.

A luciferase-based reporter phage was constructed and its utility for the rapid detection of drug resistant Mtb in a clinical setting was approved [17, 18]. However, unfortunately its application is still limited because of the unpredictability of the phage infectious rate to the clinical Mtb strains. In this study, we constructed rBCG stably expressing luciferase utilizing a stable multi-plasmid vector and a promoter that constitutively drives the transcription of the genes in Mtb, and assessed its utility for screening of TB drug candidates.

## Materials and Methods

### Construction of rBCG-MDP1-luc

Construction of pSO-luc was described previously [19]. Briefly, linker DNAs including the Shine-Dalgarno (SD) sequence (AGCTTAGTACTGGATCCGAGGACCTGCC and GATCGGCAGGTCCTCGGATCCAGTACTA) were synthesized (Sigma Genosys). pGEM-Luc (Promega, WI, USA) was digested with both BamH1 and HindIII and annealed linker DNA was inserted by ligation utilizing the ligation kit version 1 (Takara, Kyoto, Japan). The construct was then digested with HindIII and StuI and the gene fragment containing the SD sequence and the luciferase gene was inserted into pSO246 [20], which had been digested with BamH1, blunt-ended by T4 DNA polymerase, and digested with HindIII. This plasmid was designated as pSO-Luc. The promoter region of the gene encoding Mycobacterial DNA binding protein 1 (MDP1) [21] was cloned by polymerase chain reaction (PCR) using the following primers; 5’- GGGAAGCTTTCCCGATTTGGTGCATTTT and 5’- GGGGGATCCCGAAACCAGTGGTCCTCGTTTG targeting genomic DNA derived from BCG. The amplified DNA was digested with HindIII and BamHI and then inserted into the same site of pSO-Luc [20]. This recombinant BCG (rBCG) was designated as rBCG-MDP1-luc.

### Preparation of anti-mycobacterial extracts

We used the sample collection of actinomycetes strains that have been isolated from soils of various places in Japan since 1962 and kept in frozen at Institute of Microbial Chemistry (IMC) in Japan. Actinomycetes strains were cultured at 30°C for 2 days in a liquid media composed of 2% glycerol, 2% dextrin, 1% soypeptone (DIFCO), 0.3% yeast extract, 0.2% (NH_4_)_2_SO_4_, and 0.2% CaCO_3_. Then, 5% of the culture media were inoculated on the medium plates of 20 g barley in 20 ml H_2_O. The plates were incubated at 30°C for two weeks. Colonies were harvested and dissolved in 40 ml ethanol at room temperature for 24 h. Each ethanol extract (150 μl) was plated in a 96-well round bottom plate, dried completely and then dissolved in 150 μl of DMSO. These ethanol extracts derived from soil actinomycetes were designated by serial numbers.

### Bacterial culture and measurement of luciferase activity

rBCG-MDP1-luc was cultured in 7H9-ADC media containing 10 μg/ml kanamycin (KM) at 37°C until the mid-logarithmic phase. Cultures were then adjusted to an optical density of 0.1 at 600 nm and subsequently diluted 1:100 in fresh media. A 100 μl aliquot of culture was inoculated into a 96-well round bottom plate and then cultured with extracts, purified compounds, or TB drugs in a humidified incubator at 37°C under 5% CO_2_. In some experiments, the TB drugs or the purified compounds were added 2 or 3 days later. After an additional incubation, the bacterial cultures were well mixed by pipetting 30 times and 50 μl of suspension was transferred into a 96-well black flat bottom plate (Sumitomo Bakelite, Tokyo, Japan). The same volume of luciferin-ATP mixture (Promega, WI, USA) was then added into the wells and the luciferase activity was measured using a multi-plate reader ARVOsx (PerkinElmer, MA, USA).

### Susceptibility tests of Mtb strains

XDR-Mtb strains were isolated from patients in Osaka prefecture and designated as XDR-Mtb5 and XDR-Mtb44. These XDR-Mtb strains are resistant to isoniazid (INH), rifampicin (RFP), streptomycin (SM), kanamycin (KM), ethambutol (EB), levofloxacin (LV), sparfloxacin (SP), and ciprofloxacin (CPX), although they are genetically different. Susceptibility to anti-TB drugs and genetic mutations of XDR-Mtb5 and XDR-Mtb44 are shown in S1 and S2 Tables.

Mtb H37Rv, XDR-Mtb5, and XDR-Mtb44 were cultured in Mycobroth (Kyokuto, Ibaragi, Japan). Cultures were then adjusted to MacFarland 1 and diluted 1:100 in 7H9-ADC broth. A 100 μl aliquot of culture was inoculated into a 96-well round bottom plate and then cultured in the presence 1 μl of extracts for 10 to 14 days. The bacteriostatic effect was evaluated by estimating the bacterial growth visually.

### Susceptibility of effective compound to other *Mycobacterium* species and other species of bacteria

*Mycobacterium smegmatis* mc^2^155, an avirulent rapid grower of mycobacteria and *Mycobacterium avium* ATCC 25291, were cultured in 7H9-ADC. *Psudomonas fluorescens*, *Staphylococcus epidermidis*, *and Echerichia coli* were grown in LB broth. The 100 μl aliquot of bacterial suspension at OD_600_ = 0.001 was incubated in 96-well round bottom plates with two fold serial diluted active substance of 1904–1 or tetracycline. The susceptibility of drugs was assessed by macroscopic growth.

### Screening procedure

A 100 μl aliquot of rBCG-MDP1-luc suspension at OD_600_ = 0.001 was inoculated into a 96-well round bottom plate with 1 μl of the extracts. Luciferase activity was measured after 2 to 4 days incubation as described above. Macroscopic growth of BCG was checked after 9 days of incubation and designated as the 1^st^ stage of screening. Bacteriostatic extracts identified at this stage were serially two-fold diluted, added to the diluted rBCG-MDP1-luc suspension, and then cultured for 2 to 9 days. For the 2^nd^ stage of screening, the extracts still effective after a 1:1,600 fold dilution were selected. The selected extracts were then serially diluted two-fold, and subsequently added to the cultures of Mtb H37Rv, XDR-Mtb5, and XDR-Mtb44, inoculated in a 96-well round bottom plate, and cultured for 10 to 14 days. The bacteriostatic effect was evaluated by estimating the bacterial growth visually (3^rd^ stage of screening).

The extracts that were active toward Mtb strains containing XDR-Mtbs in the 3^rd^ stage of screening, were selected for the 4^th^ stage of examination. A 500 μl aliquot of extract was transferred in two sample tubes and dried, then 500 μl of ethyl acetate or n-butyl alcohol was added. After mixing, the same volume of distilled water was added in each tube and centrifuged at 20,000×g for 5 min. Each layer was separated and dried. The organic solvent layers were reconstituted with methanol, while the aqueous layers were reconstituted with methanol: water (1:1). Each layer was serially diluted two-fold and then added into rBCG-MDP1-luc in a 96-well round bottom plate (4^th^ stage of screening). The organic solvent layer containing the active substances was fractionated by high performance liquid chromatography (HPLC, Waters Separation Model 2690, MA, USA). Each fraction was again cultured with rBCG-MDP1-luc inoculated in a 96-well microplate. The active fractions were determined by measuring the luciferase activity (5^th^ stage of screening).

### HPLC analysis

The organic solvent layer containing the active substances was analyzed by HPLC using a Waters Separation Model 2690 system equipped with a C18 column (4.6 mm × 150 mm, Shiseido, Tokyo, Japan) and the flow rate was kept at 1.0 ml/min. The mobile phases were composed of 100% acetonitrile with 0.01% trifluoroacetic acid (A) and water with 0.01% trifluoroacetic acid (B). The analysis was initiated with 5% A, gradually increased to 100% over 20 min and then maintained at 100% A for 15 min. The mobile phase A was then decreased to 5% and maintained for 5 min. Fractions were collected every 20 seconds into a 96-well plate using a fraction collector CHF122SC (Advantec, Japan) and monitoring at 254 nm. Every fraction was collected in a 96-well plate, then 1 μl of each was added into the rBCG-MDP1-luc culture in a 96-well plate and cultured for 4 (for luciferase activity measurement) or 9 (for macroscopic confirmation) days. In preparation for isolating compounds on a large-scale, the mobile phases were adjusted to 50% methanol (A) and water (B).

Isolation of compounds on a large scale was achieved using a Waters 600E Multisolvent Delivery System combined with a Waters 2487 Dual λ Absorbance Detector and CHF122SC fraction collector (Advantec, Japan). Advantec CH000860 software was used to verify the compound identification. A C18 column (19 mm × 150 mm, Sunfire, MA., USA) was used as the analytical column and the flow rate was maintained at 17 ml/min. The mobile phases were composed of 30% methanol (A) and 100% methanol (B). Separation was initiated with 100% A, followed by replacement with B in a linear fashion over 25 min, and maintained at 100% B for 5 min. The fraction which showed a peak with a retention time of ~22 min was collected.

### ESI/MS

The active fraction of extract 1904–1 was analyzed by electron spray ionization mass spectrometry (ESI, Thermo Finnigan, NJ, USA). The ionization mode was positive and negative, the sheath gas (N_2_) flow rate was 30 ml/min, the spray voltage was kept at 4.5 kv, and the capillary temperature and the capillary voltage were 175°C and 25 V, respectively. For the analysis, the total ion count (full scan mode) was acquired from 150 to 2,000 Da.

### Nuclear magnetic resonance (NMR) spectroscopy

The active substance of 1904–1 was dissolved in chloroform-*d* (CDCl_3_). The nuclear magnetic resonance (NMR) spectra were recorded with a Bruker AVANCE 600 spectrometer (600 MHz for ^1^H, 150 MHz for ^13^C and 61 MHz for ^15^N). The ^1^H-NMR and ^13^C-NMR spectra were obtained at 25°C. NMR chemical shifts were referenced to the solvent peak: δ_H_ 7.26 (residual CHCl_3_) and δ_C_ 77.0 for CDCl_3_. The ^15^N chemical shift δ_N_ 75.0 of urea in dimethylsulfoxide (DMSO-d6) was used as the external standard for the ^15^N-NMR. The homonuclear two-dimensional (2D) chemical-shift-correlated spectroscopy (COSY), nuclear Overhauser enhancement spectroscopy (NOESY), rotating frame Overhauser enhancement spectroscopy (ROESY), proton detected heteronuclear 2D quantum coherence (HMQC), and heteronuclear multiple-bond correlation (HMBC) for both ^13^C and ^15^N were obtained using standard pulse sequences provided by the spectrometer manufacturer. The relaxation delay D1 was 1.5 s and a mixing time of 0.5 s were used for the NOESY experiment.

### Assessment of the cytotoxicity of the effective substance derived from extract number 1904–1

The active substance was dissolved in DMSO: cremophor: saline (1:1:8; Cremo) or DMSO: 0.1% BSA in PBS (1:9; BSA) or DMSO: saline (1:9; saline). Bone marrow derived macrophages (BMDM, 1×10^4^ cells/ml, 200 μl/well), A549 cells (human lung adenocarcinoma epithelial cell line, 1×10^5^ cells/ml, 100 μl/well) or THP1 cells (Human acute monocytic leukemia cell line) suspended in RPMI containing 10% FCS, 2 mM L-glutamine, penicillin (100 U/ml), and SM (100 μg/ml) were added in a 96-well flat bottom plate with or without various concentrations of active substance prepared as described above. Three days later, cell viability was evaluated using the commercial cell proliferation reagent WST-1 (Dojin, Kumamoto, Japan). A549 and THP1 cells were purchased from Health Science Research Resources Bank, Osaka, Japan.

### Preparation of bone marrow-derived macrophages (BMDM) and infection with Mtb

BMDM from C57BL/6 mice were prepared utilizing L929-cell conditioned medium (LCCM) as a source of macrophage colony stimulating factor. The cells were cultured in DMEM media supplemented with 10% FCS, 20% LCCM, 10 mM HEPES, 100 U/ml penicillin, and 100 μg/ml SM (BMDM culture media) for 3 days at 37°C in 5% CO_2_ in bacterial Petri dishes. Three days after seeding the cells, the media was exchanged for fresh BMDM media and incubated for a further 4 days. After washing with PBS once, adherent macrophages were removed by treatment with a trypsin/ EDTA solution (0.5 mg/ml trypsin and 0.2 mg/ml EDTA in PBS).

BMDM were seeded in a 96-well tissue culture plate (5×10^4^ cells/well) and were cultured for 1 day under 5% CO_2_. Then the cells were infected with Mtb H37Rv at a multiplicity of infection (MOI) of 1:1 for 12 h in DEMEM supplemented with 1% FCS in the absence of antibiotics. Cells were washed with DMEM containing 10% FCS three times and then cultured with or without active substance in DMEM containing 10% FCS and 50 μg/ml of gentamycin. Seven days after cultivation, cells were lysed by adding 1/10 volume of PBS containing 5% Triton X. Aliquots of cultures were inoculated on 7H11 agar containing 10% OADC and 10 μg/ml of KM. Colony forming units (CFU) were counted after 4 weeks of culture at 37°C.

THP1 cells were seeded (5×10^4^ cells/well) in 96-well plate with DMEM supplemented with 10% FCS, 100 U/ml penicillin, 100 μg/ml SM, and 100 nM PMA (Sigma) for 1 day. PMA was then removed by washing the wells by phosphate buffer. Cells were then incubated in the media without PMA for 2 days and infected with Mtb H37Rv as described above. After the infection, cells were cultured with or without active substance for seven days.

### Isolation of resistant strains against the active substance derived from extract 1904–1

When the rBCG-MDP1-luc culture reached an OD of 0.1, the active substance was added at a concentration of 10 μg/ml. Four days after cultivation, bacterial cultures were diluted to an OD of 0.02 and subsequently inoculated into 7H11 agar containing the active substance at various concentrations. The obtained colonies were inoculated into 7H9-ADC media without active substance and then they were inoculated on 7H11 agar with or without active substance again. The colonies which grew on the agar containing 0.625 to 1.25 μg/ml active substance were selected.

### Whole genome sequencing of resistant BCG against active substance derived from extract 1904–1

DNA was extracted using a genomic DNA extraction kit (QIAGEN, Venlo, Netherlands). Genomic DNAs were sequenced using a 454 GS-FLX Titanium Sequencer and the reads were assembled using Newbler version 2.3 and then analyzed by Genome traveler ver. 2.1 (In Silico Biology Inc., Yokohama, Japan).

## Results

### Kinetics of CFU and luciferase activity during culture of rBCG-MDP1-luc

**Fig 1:**
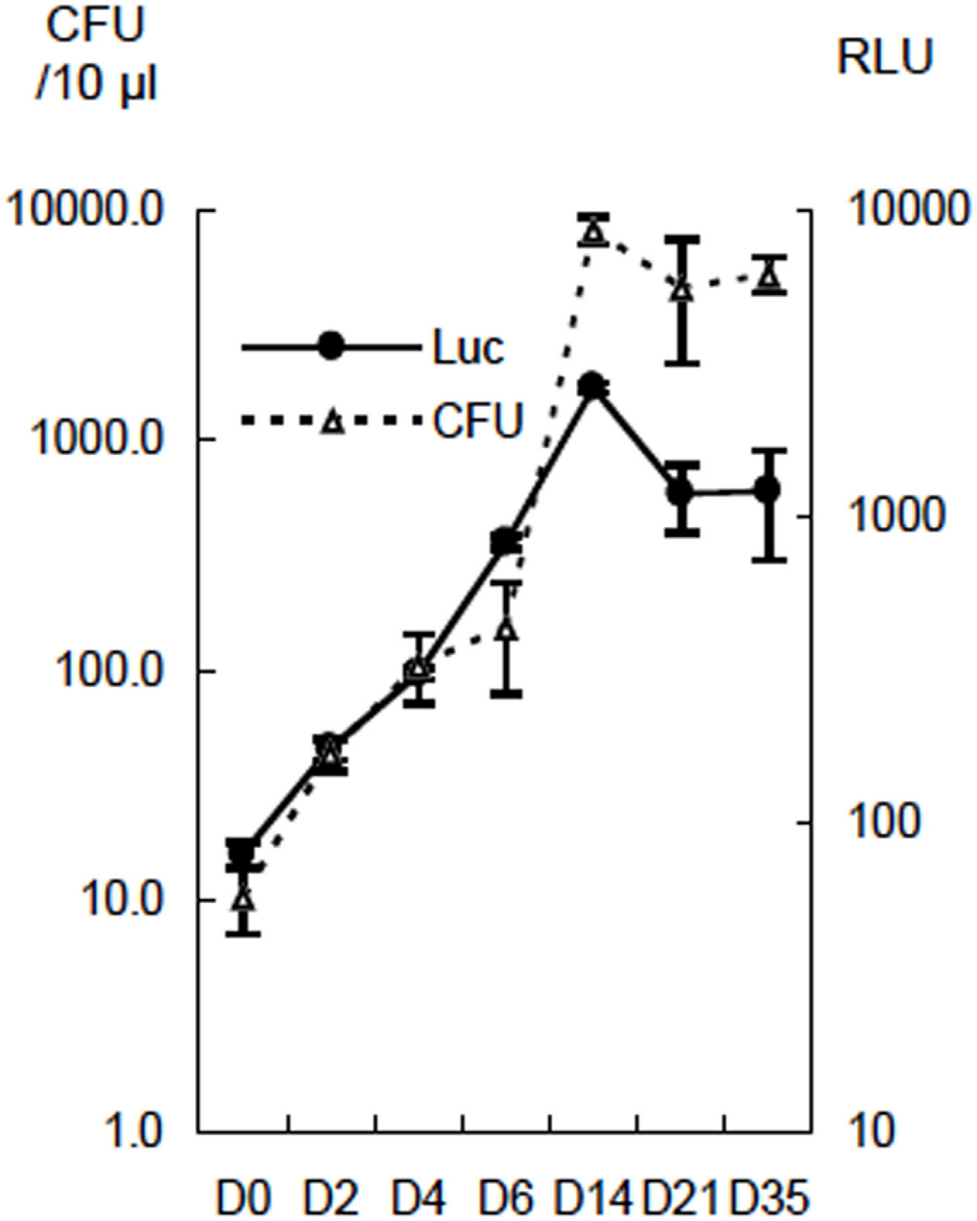
Correlation between luciferase activity and the CFU of rBCG-MDP1-luc. A bacterial suspension of rBCG-MDP1-luc adjusted to an optical density (OD) = 0.001 at 600 nm in 7H9-ADC media containing 10 μg/ml Km was incubated in a 96-well round bottom plate at 37°C. At each time point, 50 μl of bacterial culture was mixed with the same volume of luciferin-ATP mixture (Promega) and the relative light units (RLU) of luciferase was immediately determined using a luminometer. At the same time, bacteria were inoculated on 7H11-OADC agar containing 10 μg/ml of Km after serial dilution. Three weeks after cultivation, the CFU were counted and compared with the corresponding control. The representative data of two independent experiments are presented as the means ± SD.

To establish a rapid drug screening method, we first constructed rBCG expressing luciferase driven by the promoter of the MDP1/HupB gene, one of essential genes in Mtb. This recombinant BCG was designated as rBCG-MDP1-luc[22, 23]. We cultured rBCG-MDP1-luc then compared the relative light units (RLU) of luciferase activity and CFU. As shown in Fig 1, after inoculation of 100 μl of the rBCG-MDP1-luc culture at 0.001 OD_600_ in a 96-well plate, the luciferase activity was increased until 14 days where it reached a plateau and then gradually decreased. The RLU was detected for concentrations over 10^3^ CFU/ml of rBCG-MDP1-luc and was closely correlated with CFU as described previously [18].

### RLU and CFU responses of rBCG-MDP1-luc to anti-TB drugs

**Fig 2:**
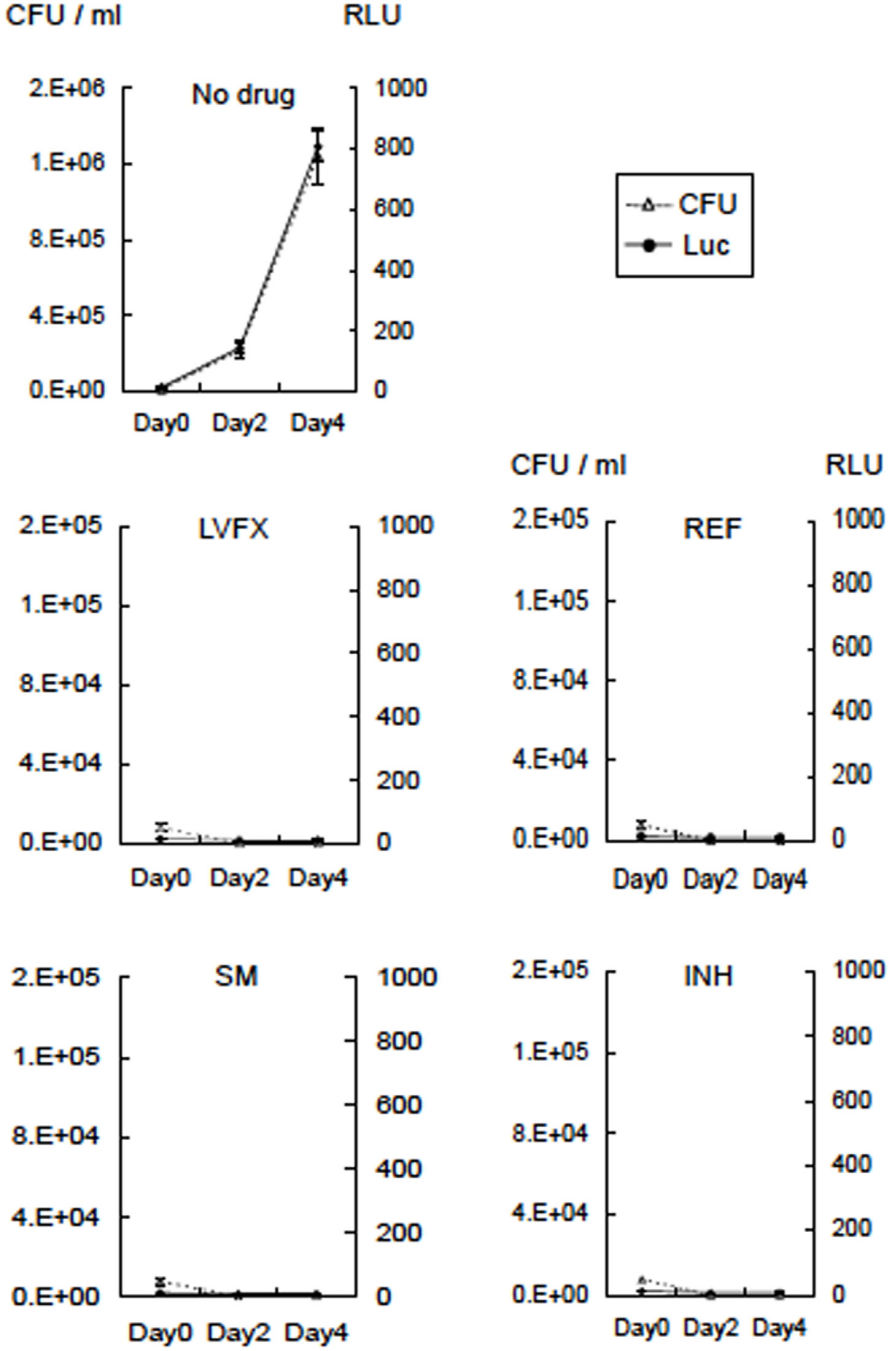
TB drugs abrogate the increase of both luciferase activity and the CFU of rBCG-MDP1-luc. TB drugs, such as INH, RFP, LVFX, and SM, were added to the bacterial suspension at a concentration of 0.5 μg/ml, 8 μg/ml, 1 μg/ml, and 4 μg/ml, respectively (10 fold concentration of the MIC of each drug). At each time point, 50 μl of bacterial suspension was mixed with the same volume of the luciferin-ATP mixture (Promega) and then the luciferase activity was determined immediately as the RLU. At the same time, each bacterial culture was serially diluted and inoculated on 7H11-OADC agar containing 10 μg/ml of Km. Three weeks after cultivation at 37°C, the CFU were counted and compared with the RLU. The representative data of two independent experiments are presented as the means ± SD.

We assessed the effectiveness of the currently available TB drugs, such as LVFX, RFP, SM, or INH, on the luciferase activity of rBCG-MDP1-luc. We added TB drugs to the culture and monitored the growth for the following 96 h. The luciferase activity of rBCG-MDP1-luc treated with any of the drugs did not increase, while the non-treated culture showed a rapid increase in activity during the 4 days of cultivation. The number of viable bacteria (CFU) counted after 3 to 4 weeks of culture on the 7H11-OADC agar coincided with the luciferase activity (Fig 2).

**Fig 3:**
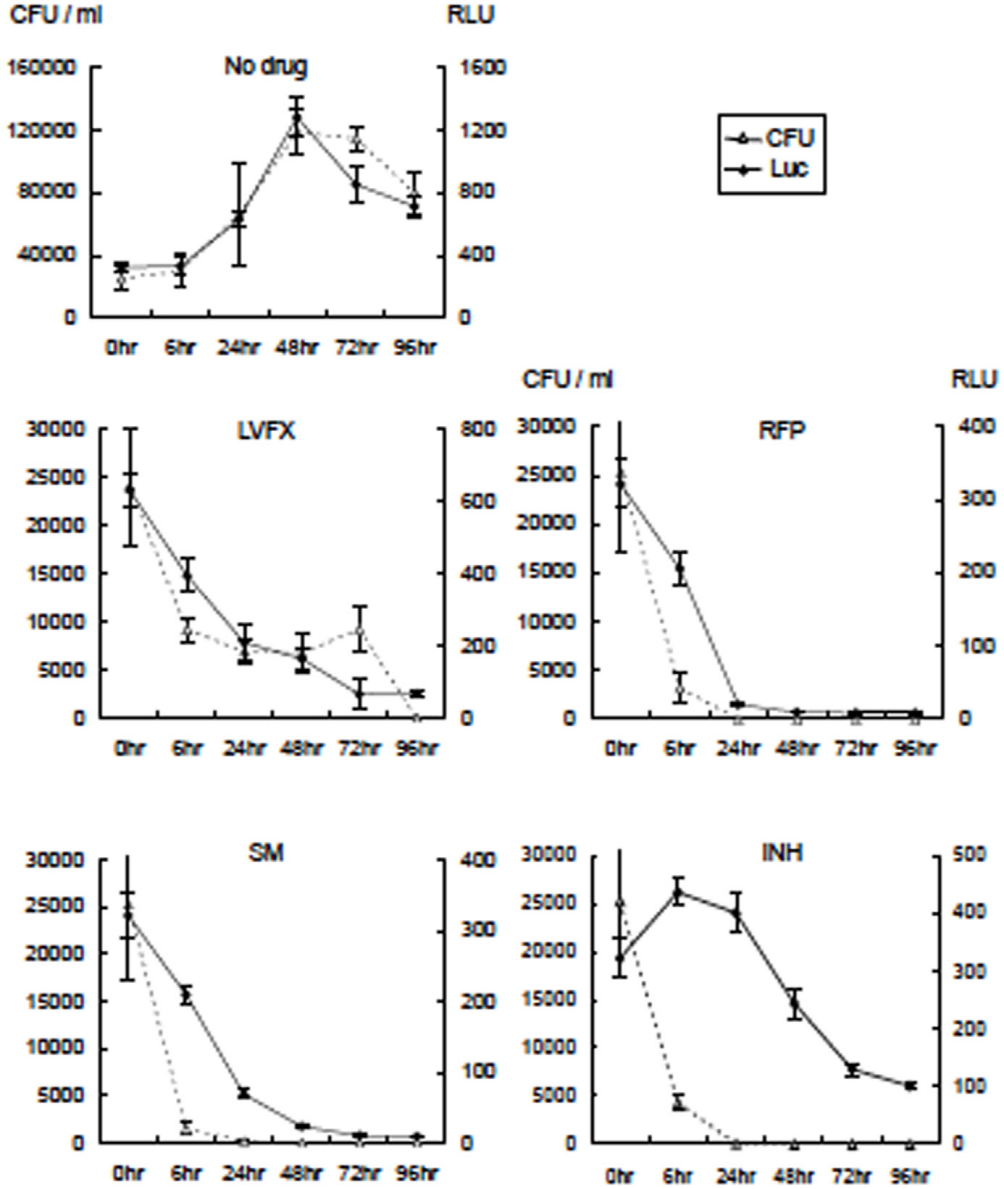
Kinetics of luciferase activity reduction after treatment with TB drugs. Three days after culturing rBCG-MDP1-luc, TB drugs were added at the same concentrations as depicted in Fig 2. At each time point, 50 μl of bacterial suspension were mixed with the same volume of luciferin-ATP mixture (Promega) and the luciferase activity was determined immediately as the RLU. At the same time, the bacterial culture was serially diluted and inoculated on 7H11-OADC agar containing 10 μg/ml of Km. Three weeks after cultivation at 37°C, the CFU were counted and compared with the RLU. The representative data of two independent experiments are presented as the means ±SD.

Next, we examined the kinetics of the luciferase activity after drug treatment. After the rBCG-MDP1-luc was grown on the culture plate, each drug was added and the luciferase activity was monitored for the following 96 h. All treated cultures were found to have both decreased luciferase activity and CFUs with some variations (Fig 3). In the case of LVFX treatment, which inhibits DNA synthesis, the reduction of luciferase activity was closely correlated with that of the CFU. By comparison, the reduction of luciferase activity after treatment with RFP and SM was rapid but relatively retarded from that of the CFUs. RFP and SM are known transcription and translation inhibitors, respectively. By contrast, after treatment with INH, which inhibits cell wall synthesis, the luciferase activity was significantly retarded compared with the decrease in the CFUs and remained high even 96 h after the treatment (Fig 3).

Typical results are shown for ethambutol, another inhibitor of cell wall biogenesis. A reduction in luciferase activity apparently resulted from the decrease in the CFU even 96 h after treatment (data not shown). Most notably, the luciferase-based assay system can detect the effect of all types of TB drugs within 2 to 4 days. It is more sensitive for detecting compounds which inhibit replication, transcription, and translation than those which inhibit cell wall biosynthesis.

### Assessment of rBCG-MDP1-luc for TB drug screening

**Fig 4:**
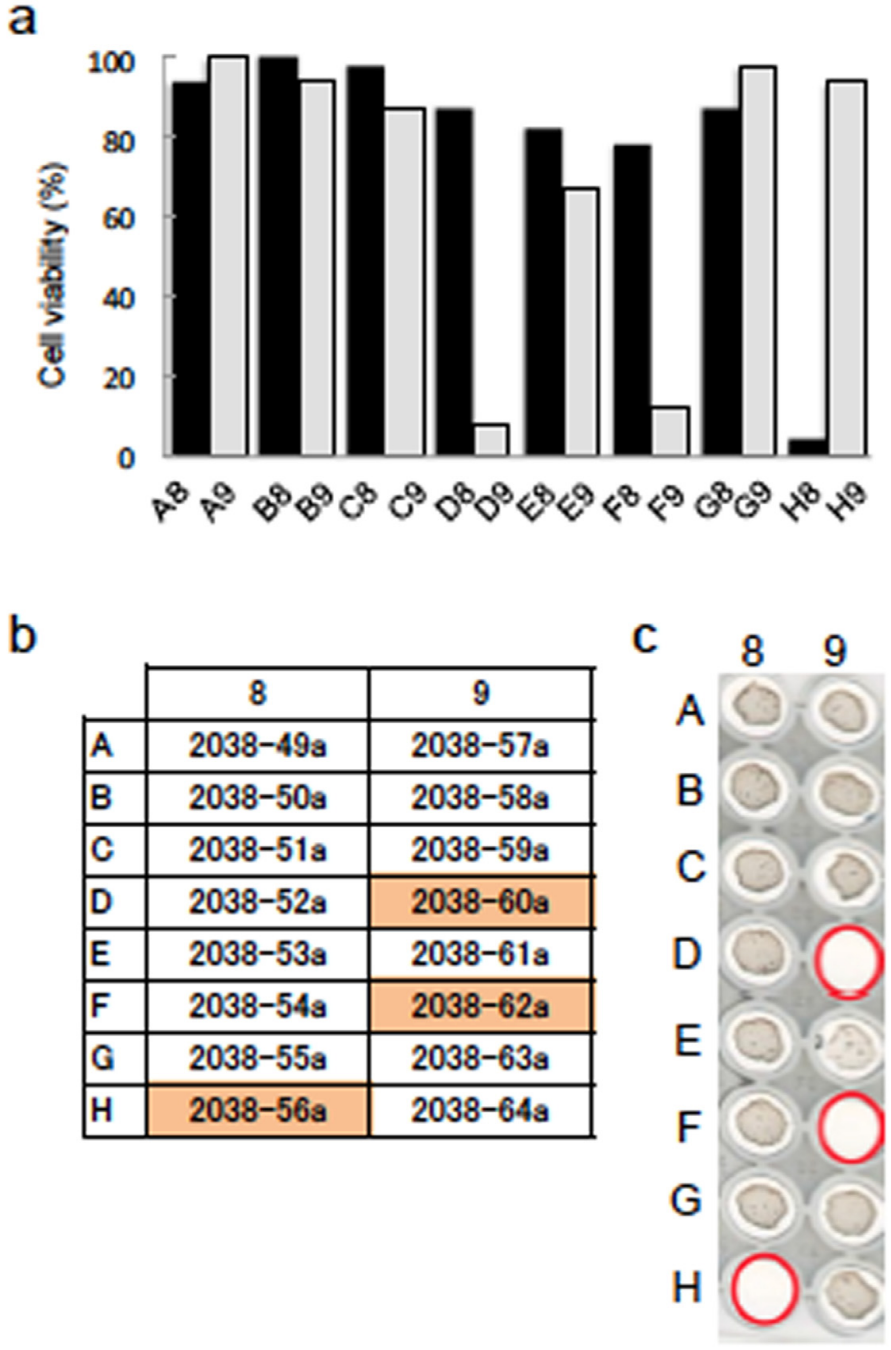
Pilot screening of actinomycetes-derived extracts using rBCG-MDP1-luc (^1st^ stage of screening). rBCG-MDP1-luc was cultured in the presence or absence of 1 μl of each actinomycetes extract dissolved in DMSO in a 96-well round bottom plate. (a) Four days after cultivation, 50 μl of bacterial culture was mixed with the same volume of luciferin-ATP mixture and the luciferase activity was measured. The data were normalized against positive control (DMSO). b) The list of extracts numbers is described and the effective extracts are noted in orange. (c) Nine days after incubation, the plate was scanned to observe the macroscopic growth of rBCG-MDP1-luc. Wells where growth inhibition was observed were marked by red circles. The representative data of three independent experiments are presented (a-c).

Herein, we have examined whether rBCG-MDP1-luc can be applied for screening of active compounds against TB. To date, over 80% of antibiotics have been obtained from actinomycetes and thus, we tested the activity of a crude extract isolated from actinomycetes. As mentioned above, 100 μl of a rBCG-MDP1-luc culture at an OD_600_ of 0.001 was incubated with 1 μl of the extracted compound in a 96-well plate for 4 days to investigate its bactericidal ability by measuring luciferase activity, in conjunction with a replica plate incubated for 9 to 14 days to macroscopically check bacterial growth. We observed that 3 extracts, designated as 2038-56a, 2038-60a, and 2038-62a, were classified as growth suppressive agents by quantification of their luciferase activities (Fig 4a) in agreement with the results obtained from the macroscopic examination (Fig 4b and 4c, shown in red).

**Fig 5:**
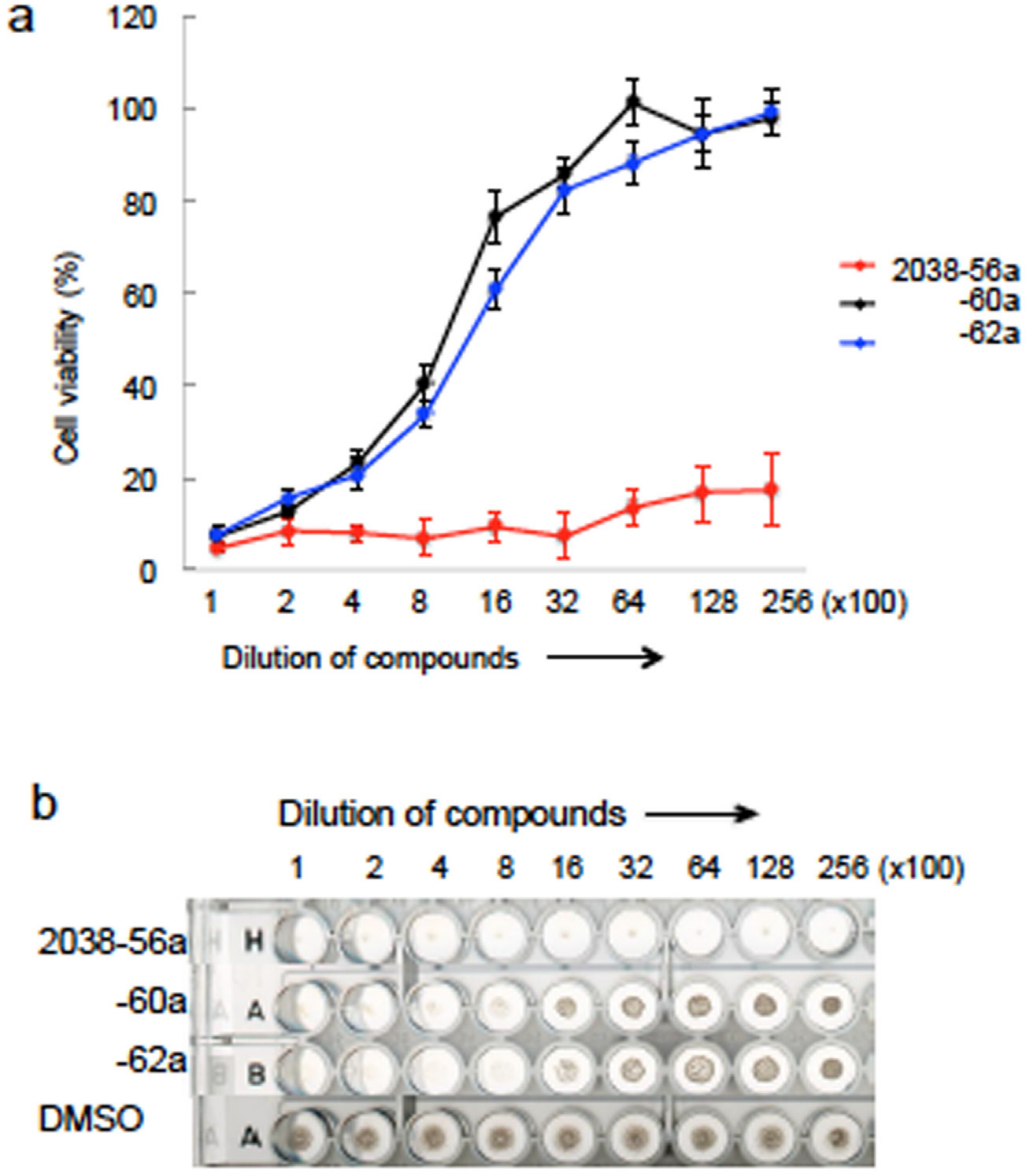
Pilot screening of actinomycetes-derived extracts using rBCG-MDP1-luc (^2nd^ stage of screening). rBCG-MDP1-luc was cultured in media containing 1 μl of a two-fold serially diluted extract in a 96-well round bottom plate. (a) Four days after cultivation, 50 μl of bacterial culture was mixed with the same volume of luciferin-ATP mixture and the luciferase activity was measured. The data were normalized against DMSO. The representative data of two independent experiments are presented as the means ±SD. (b) Nine days after incubation, the plate was scanned to observe the macroscopic growth of rBCG-MDP1-luc. (a and b) Extract 2038-56a was effective even after a 1:25,600 dilution.

We next assessed the dilution effect of these 3 growth suppressive extracts on luciferase activity. Extract 2038-56a was the most effective as it suppressed bacterial growth at dilutions up to 1:25,600 while extracts 2038-60a and 2038-62a suppressed bacterial growth less effectively (Fig 5). The luciferase activities of the rBCG-MDP1-luc were again in agreement with those from observation of the macroscopic growth of bacteria after treated with the extracts of actinomycetes.

### TB-drug screening of 10,080 extracts of actinomycetes

We next used rBCG-MDP1-luc to screen 10,080 extracts from actinomycetes for possible TB drugs. A total of 609 extracts possessed a bactericidal effect at a 1:100 dilution (1^st^ stage of screening). Then we performed a similar assessment after diluting the effective extracts, and 137 were found to be effective after dilutions of up to 1:1600 (2^nd^ stage of screening).

Further screening of the 137 identified extracts was conducted. Their effect on 3 Mtb strains, the laboratory Mtb strain H37Rv and 2 clinical XDR-Mtb strains, was examined (3^rd^ stage of screening). The results showed that 41 extracts were effective against H37Rv and at least one of XDR-Mtb strains, even when the extracts were diluted more than 1:200 (S3 Table).

**Fig 6:**
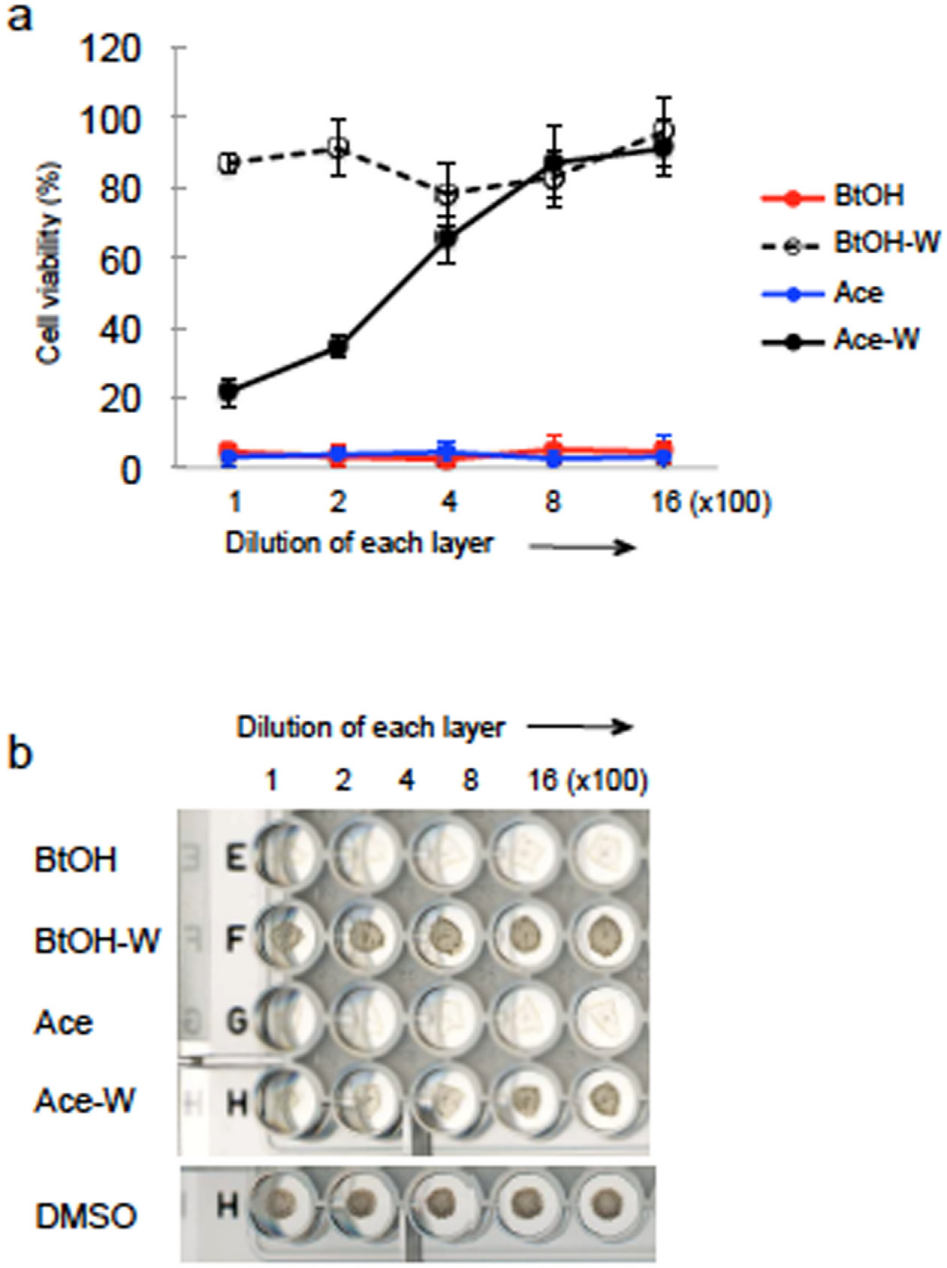
Two-layer distribution test of active 1904–1 extract (^4th^ stage of screening). The 1904–1 extract was fractionated into a two-layer distribution using acetic ethyl and water, or n-butyl alcohol and water. Extracts of each layer were dried and reconstituted in methanol for the ethyl layer and n-butyl layer and in 50% methanol for the water layer. rBCG-MDP1-luc was cultured in the presence or absence of 1 μl of a two-fold serially diluted layered extract. Luciferase activity (a) and macroscopic growth (b) were checked after 96 h and 9 days cultivation, respectively. BtOH: n-butyl alcohol layer, BtOH-W: n-butyl alcohol-water layer, Ace: acetic ethyl layer, Ace-W: acetic ethyl-water layer. The data were normalized against DMSO control and presented as mean ± SD. The representative data of two independent experiments are shown (a and b).

A two-layer distribution test using distilled water and ethyl acetate or distilled water and n-butyl alcohol for these 41 compounds was completed. As a 4^th^ stage of screening, rBCG-MDP1-luc was cultured with each diluted layer of extract. Thirty nine compounds contained in the organic solvent layers possessed bactericidal activity. An example is shown in Fig 6. Both the ethyl acetate and n-butyl alcohol layers (Ace, BtOH) but not the water layer (Ace-W or BtOH-W) significantly suppressed the luciferase activity of rBCG-MDP1-luc (Fig 6a). Some of results were confirmed by examining the macroscopic bacterial growth and in all cases, the results aligned with the measurement of luciferase activity (Fig 6b).

A total of 18 extracts possessed growth suppressive activity, even after a 6,400 fold dilution (2^nd^ stage of screening) and were also active against the Mtb strains. We next examined the cytotoxicity of these remarkably effective 18 extracts to the eukaryotic cells (S3 Table). The data showed that 17 had more than 75% of cytotoxicity to either L1210 cells (mouse lymphocytic leukemia cells) or A549 cells (human lung adenocarcinoma epithelial cells) and 9 of them contained well-known antibiotics, such as actinoleukin, actinomycin, and hygromycin. However, the 1904–1 extract was less cytotoxic to the cells (39% cell death), did not contain known antibiotics, and was effective at dilutions up to 25,600 fold. Based on these results, we selected extract 1904–1 for further characterization as a TB drug candidate.

### Taxonomy of the producing strain

Compound 1904 was a crude product of strain MM334-153F1 isolated from a soil sample collected at Shinagawa-ku, Tokyo, Japan. Strain MM334-153F1 formed well-branched substrate mycelia and straight to flexuous aerial mycelia. The type of diaminopimelic acid isomers in whole-cell hydrolysates of strain MM334-153F1 was determined to be the LL-form by the method of Staneck and Roberts [24]. The partial 16S ribosomal RNA gene sequence (1399 bp) was determined. The strain showed high similarity with *Streptomyces lanatus* (NBRC 12787^T^, 1381/1400 bp, T: Type strain, 98.6%), *S*. *rameus* (NBRC 3782^T^, 1376/1399 bp, 98.3%) and *S*. *coacervatus* (IFM 11055^T^, 1374/1399 bp, 98.2%) [24]. These phenotypic and genotypic data suggested that strain MM334-153F1 belongs to the genus *Streptomyces*. Therefore, the strain was tentatively designated as *Streptomyces* sp. MM334-153F1.

### Purification and MS analysis of the active substance of 1904–1

**Fig 7:**
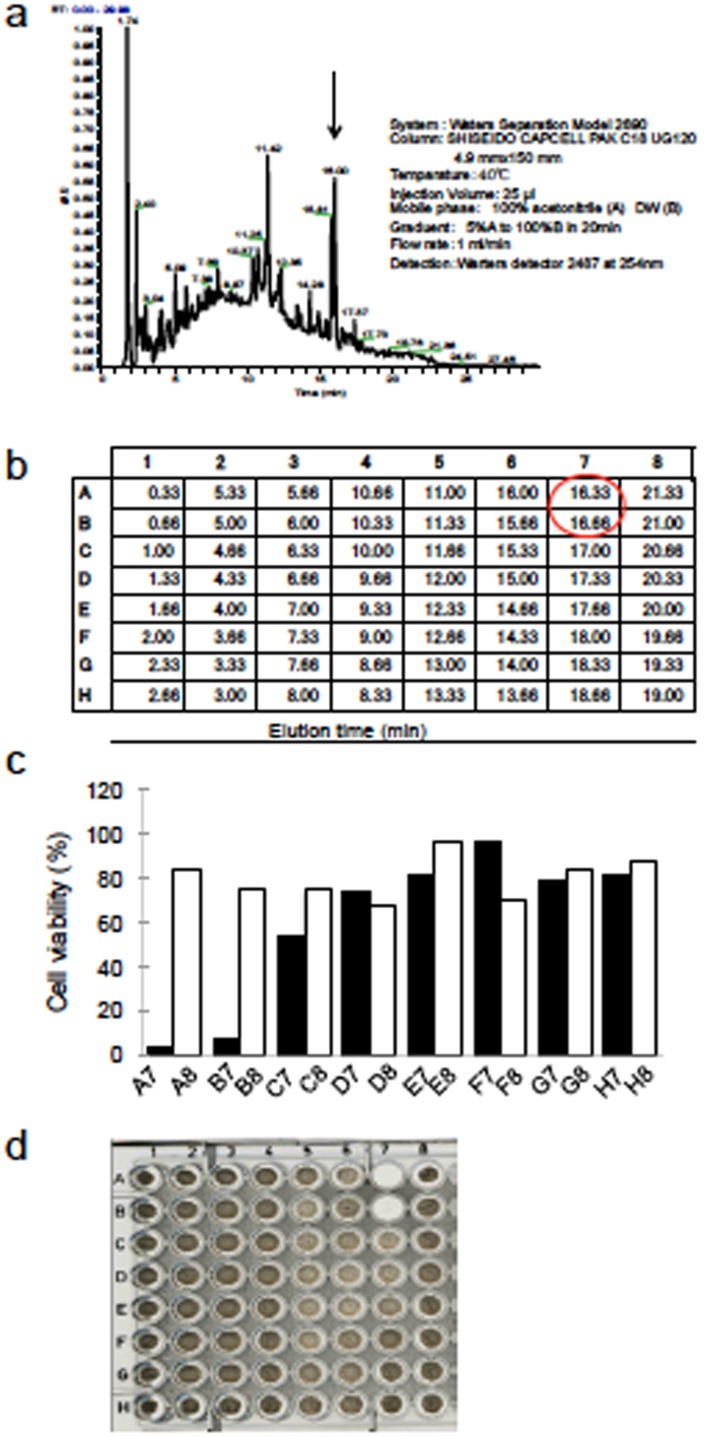
Separation of the 1904–1 extract by HPLC. The BtOH layer of the 1904–1 extract was separated by HPLC using a C18 column as described in Material and Methods. A brief method is described on the right side of the chromatogram of (a). (a) UV spectroscopic chromatogram at 254 nm. (b) The retention times of the eluents. Each eluent was incubated with rBCG-MDP1-luc and their activities were obtained by luciferase activity measurement (c) and macroscopic assay (d) 4 and 9 days after incubation, respectively. The peak of the active substance is indicated by the arrow in (a) and its retention times are marked by red in (b). The time of elution is delayed from that of the peak in the chromatogram because of a time lag (a and b). The data were normalized against DMSO control (c). All data of three independent experiments are presented (a-d).

**Fig 8:**
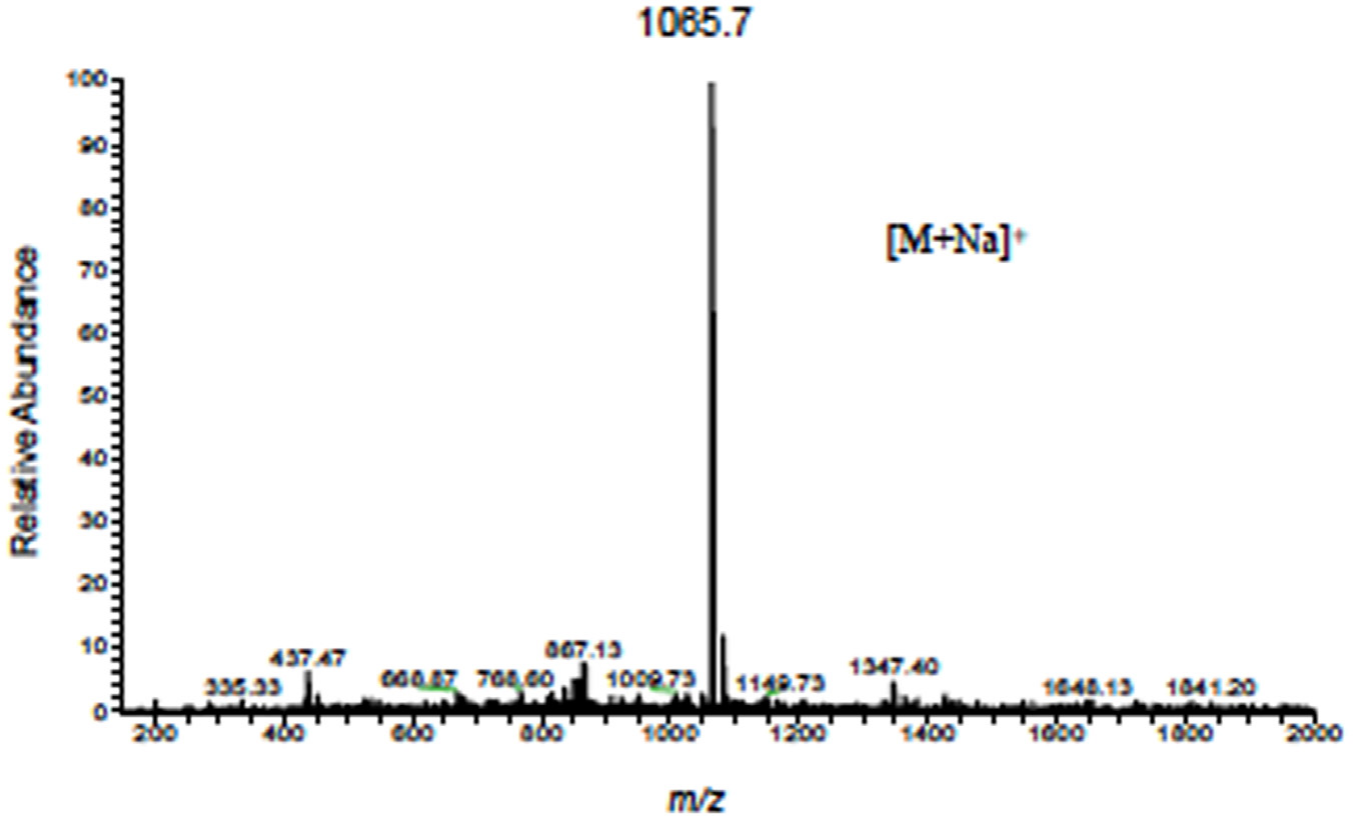
Electrospray mass spectrometry (ESI)/MS data of the active fractions of the 1904–1 extract. Effective fractions of the 1904–1 extract obtained by HPLC were analyzed by ESI-mass spectrometry using the positive ion mode.

We fractionated the BtOH fraction of the 1904–1 extract by HPLC using a C18 column as shown in Fig 7a. The active substance had a peak around 16 minutes, a retention time equivalent to a fraction eluted by 19% of water (Fig 7a and 7b). As shown by the results of the luciferase activity measurements and macroscopic examination, in the presence of the active substance, the growth in 2 wells was inhibited (Fig 7c and 7d). The molecular weight of this active substance was determined by ESI/MS as 1,042 based on its *m/z* value (Fig 8).

### Large-scale purification of the active substance of the 1904–1 extract

For further characterization of the 1904–1 extract active substance, we established a large-scale and cost-effective HPLC purification system. We replaced acetonitrile in the mobile phase with methanol. The active fractions were observed in peaks at retention times of 18.33 to 19.33 minutes (S1 Fig). Then we purified large amounts of active substance from the 1904–1 extract using a Waters 600E Multisolvent Delivery large-scale purification system (Waters, MA, USA). We confirmed purification of the active substance by this system with ESI/MS (S2 Fig).

### Determination of the MIC values of the active substance of 1904–1 extract

**Fig 9:**
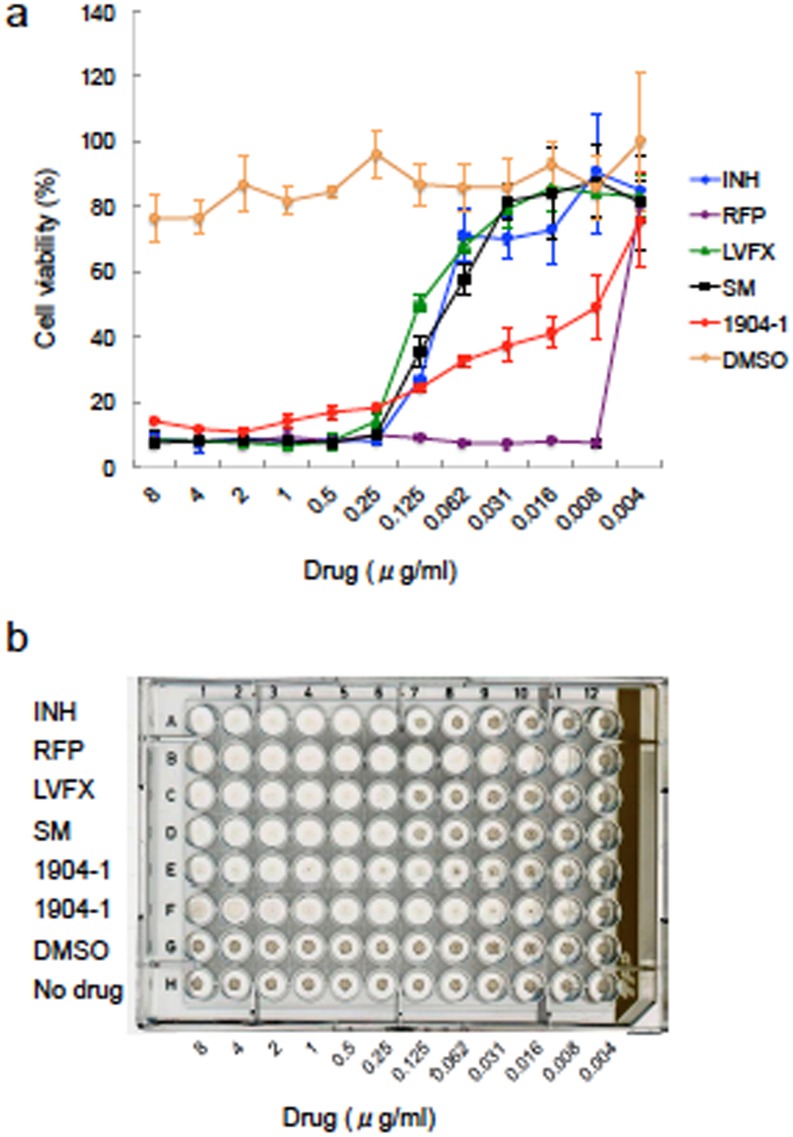
Comparison of the efficacy of the active substance of the 1904–1 extract with commercially available TB drugs. rBCG-MDP1-luc was cultured in the presence of various concentrations of LVFX, REF, SM, INH, and the active substance of the 1904–1 extract. (a) Luciferase activity was measured 4 days after cultivation. The data were normalized against medium alone. (b) Nine days after incubation, the plate was scanned to see the macroscopic growth of rBCG-MDP1-luc. The representative data of three independent experiments are presented (a and b).

Utilizing the purified active substance from the 1904–1 extract, we evaluated its bactericidal activity against rBCG-MDP1-luc and compared it with those of currently available anti-TB drugs. As shown in Fig 9, the MIC of the active substance from the 1904–1 extract was 0.125 μg/ml, which was more effective than SM and LVFX and similar to that of INH (Fig 9).

We also determined the MIC of the active substance against Mtb H37Rv and the two clinical XDR-Mtb strains, XDR-Mtb5 and XDR-Mtb44. The MIC against Mtb H37Rv, XDR-Mtb5, and XDR-Mtb44 was 0.5, 2.0 and 7.5 μg/ml, respectively. The active substance was also effective to Mtb H37Rv in the stationary phase (S3 Fig).

**Fig 10:**
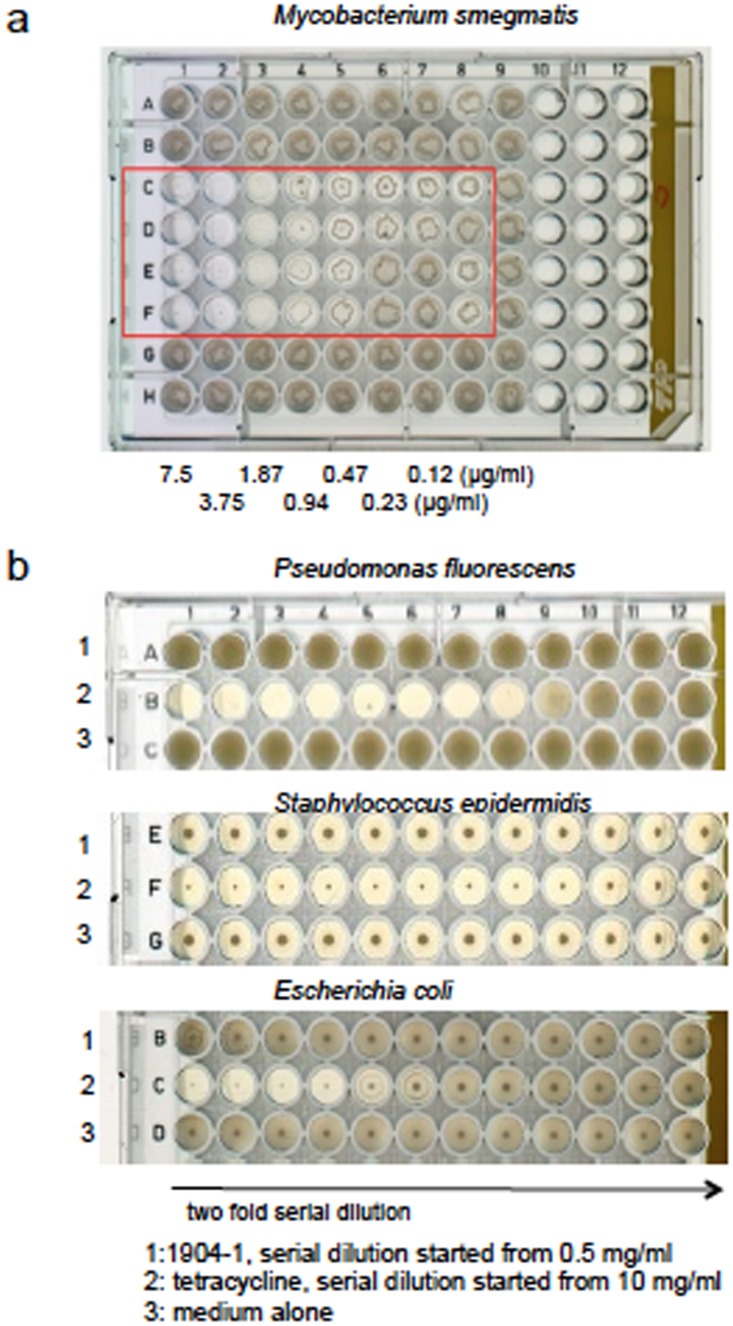
Species-specific efficacy of the active substance of the 1904–1 extract. Efficacy of species-specificities of the active substance of the 1904–1 extract was studied. *M*. *smegmatis* was cultured in the media containing serial dilutions of the 1904-1-derived active substance. (a) Four days after, the macroscopic growth was scanned. (b) Similarly, *Pseudomonas fluorescens*, *Staphylococcus epidermidis*, and *Escherichia coli* were cultured in the presence of the 1904-1-derived active substance, tetracycline, or in the absence of antibiotics for 1 day?. The active substance of the 1904–1 extract and tetracycline were two-fold serially diluted from 0.5 μg/ml and 10 μg/ml, respectively. The macroscopic growth of the bacteria was scanned. The representative data of two independent experiments are presented.

The potency of the active substance from the 1904–1 extract against other bacterial species was also examined. It was effective against another species of *Mycobacterium*, *Mycobacterium smegmatis*, with a MIC value of 0.47 μg/ml by macroscopic bacterial growth examination (Fig 10a). In contrast, the active substance of 1904–1 extract did not suppress the growth of *Mycobacterium avium* (data not shown). Other species of bacteria such as *Pseudomonas fluorescens*, *Staphylococcus epidermidis*, or *Escherichia coli* were resistant to the active substance of 1904–1 (Fig 10b). These results suggested that the bactericidal effect of active substance of 1904–1 is limited to some species of *Mycobacterium*.

### The active substance of the 1904–1 extract is effective against intracellular Mtb

**Fig 11:**
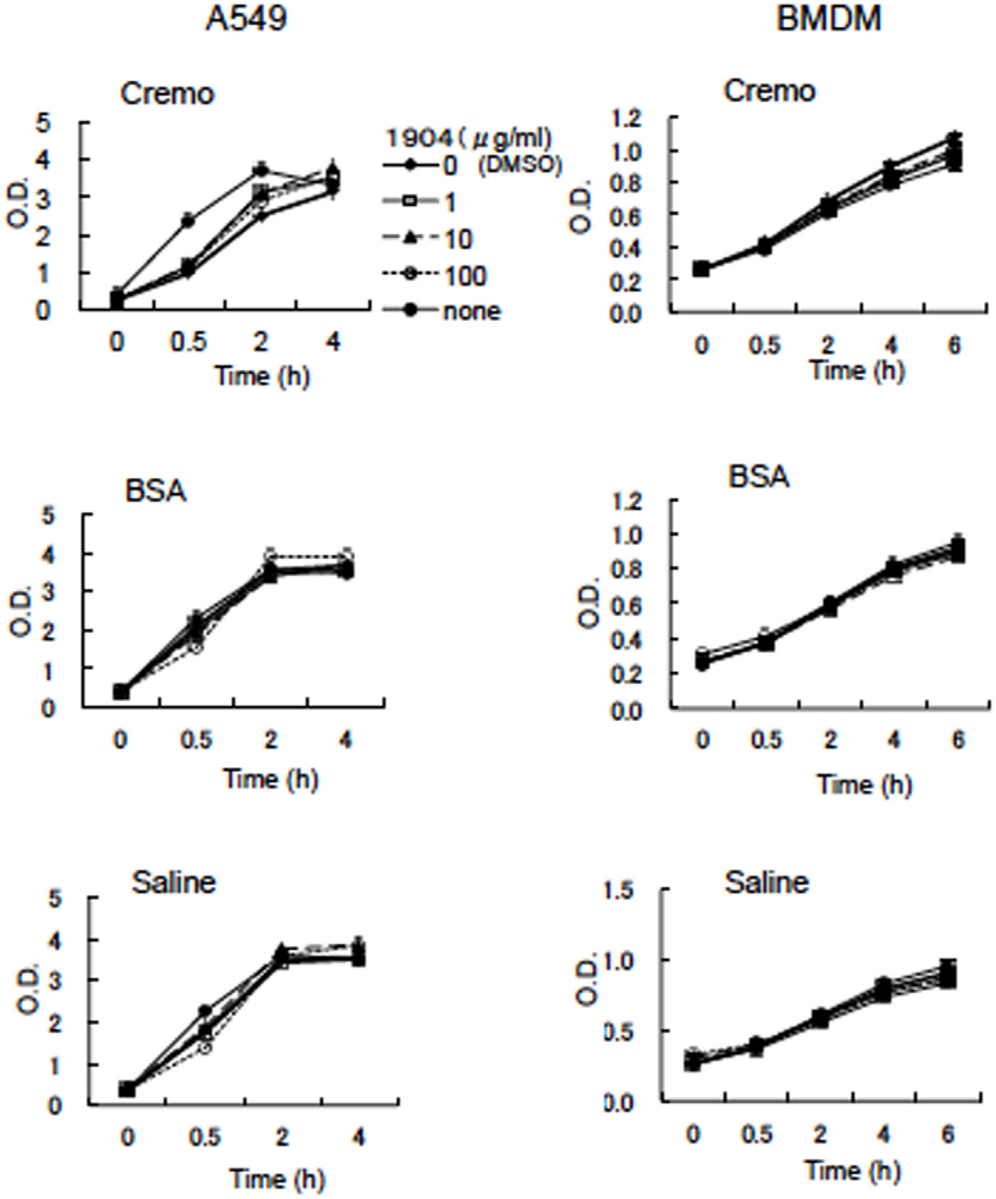
Analysis of the cytotoxicity of the 1904-1-derived active substance toward eukaryotic cells. The active substance of the 1904–1 extract was dissolved in saline containing 10% cremophor EL (Cremo), 0.01% BSA (BSA), or saline. Ten-fold serial dilutions of the active substance were added to the culture of both A549 cells and bone marrow derived macrophages of mice (BMDM), seeded at 1×10^4^ cells/100 μl media on a 96-well plate. Three days after cultivation, cell viability (respiration) was checked using a cell viability detection kit. Increases in respiration were monitored at 0, 0.5, 2, 4 and 6 (only for BMDM) h after adding the redox-sensitive growth indicator. The representative data of two independent experiments are presented.

Mtb is an intracellular pathogen and can replicate in macrophages. Therefore, we tested the bactericidal effect of the 1904-1-derived active substance against Mtb in infected macrophages. Before determining this efficacy, we examined the cytotoxic effect of the active substance of the 1904–1 extract. It permitted the normal growth of mouse bone marrow derived-macrophages of mice (BMDM) and the human lung adenocarcinoma epithelial cell line, A549, even at 100 μg/ml dissolved in Cremophor EL containing 0.01% BSA and 150 mM NaCl (Fig 11). However, it was relatively toxic to the human monocytic cell line, THP1, suppressing its multiplication at concentrations more than 1 μg/ml (S4 Fig).

**Fig 12:**
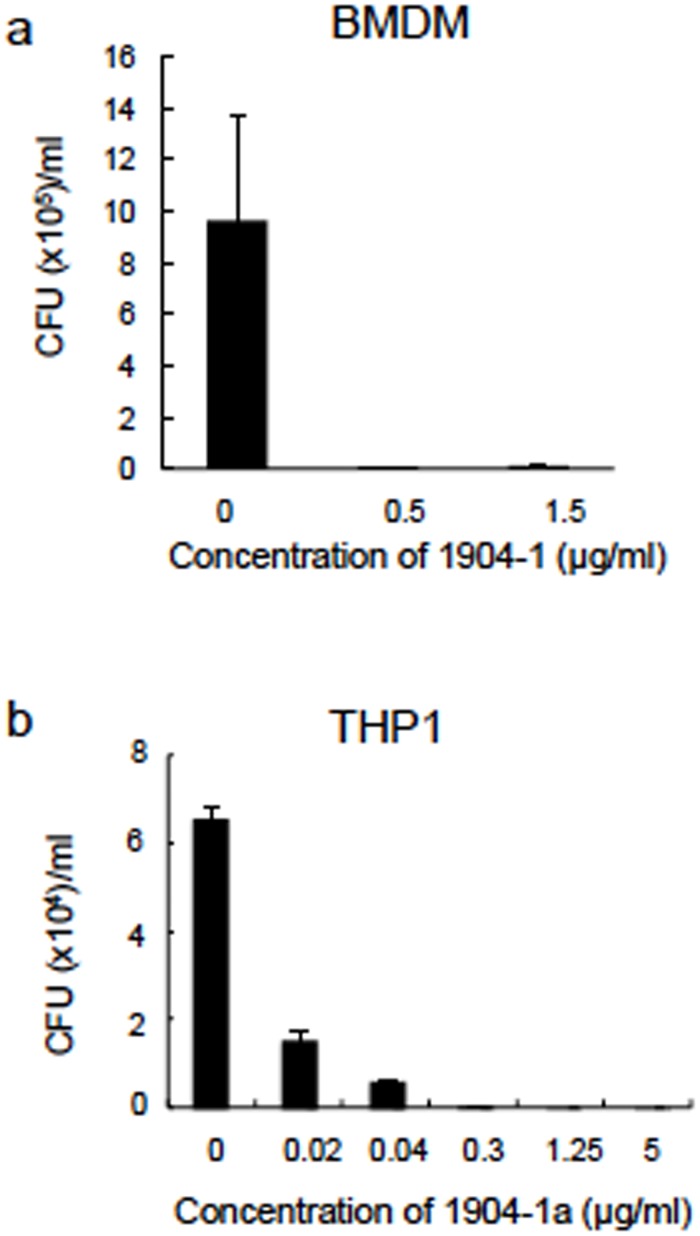
Bactericidal effect of the 1904-1-derived active substance against intracellular Mtb. In vitro-cultured 5×10^4^ cells/well of mouse bone marrow derived macrophages (BMDM) (a) or THP1 cells (b) were infected with Mtb H37Rv at a MOI of 1:1 for 12 h. After washing the uninfected bacteria, cells were cultured in the presence or absence of the active substance of 1904–1 at several concentrations as indicated in the graphs. Seven days after cultivation, cells were lysed and aliquots of culture were inoculated on 7H11-OADC agar to count the living bacteria. The CFU was counted after 4 weeks. The representative data of two independent experiments are presented as means ± SD.

Then we infected both BMDM and THP1-derived macrophages with Mtb H37Rv. After washing the uninfected bacteria, we added the active substance from the 1904–1 extract and studied its efficacy by determining the CFU after 6 days of treatment. The active substance suppressed Mtb growth at 0.5 μg/ml and 0.02 μg/ml in BMDM and THP1-derived macrophages, respectively (Fig 12). These results suggested that the active substance of 1904–1 is also effective against intracellular Mtb.

### Analysis of the chemical structure of the active substance of 1904–1 by NMR

**Fig 13:**
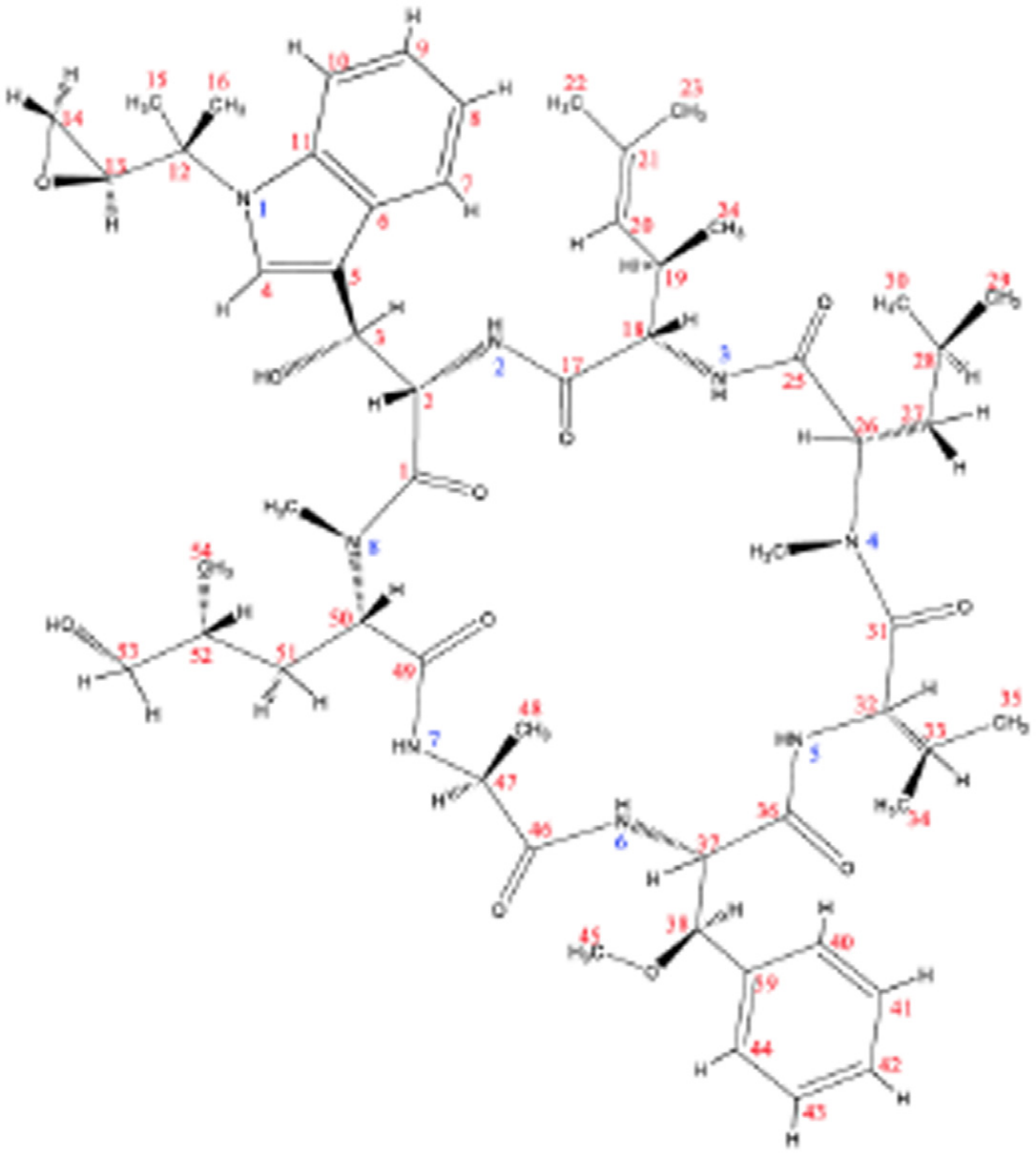
Chemical structure of the active substance of 1904–1 determined by NMR. The active substance of the 1904–1 extract was dissolved in chloroform-d (CDCl_3_) and NMR spectra were recorded with a Bruker AVANCE 600 spectrometer (600 MHz for ^1^H, 150 MHz for ^13^C and 61 MHz for ^15^N).

We obtained ^1^H and ^13^C NMR spectra to determine the chemical structure for the molecular formula C_56_H_82_N_8_O_11_ based on ESI/MS data. The proton detected heteronuclear 2D quantum coherence (HMQC) spectrum, and the heteronuclear multiple-bond correlation (HMBC) spectrum for ^15^N revealed eight nitrogenous correlation signals indicating that the active substance of 1904–1 possessed 8 amino groups, three of them were secondary amines, and the others were tertiary amines. Complete assignments for the ^1^H and ^13^C-NMR signals in CDCl_3_ were accomplished using a combination of 2D NMR, including ^1^H-^1^H COSY, HMQC, and HMBC. Comparison of the ^1^H- and ^13^C-NMR data of the active substance with those of cyclomarin A led to confirmation of its identification as cyclomarin A, which recently was reported to show an anti-mycobacterial inhibition effect (Table 1) [25, 26]. The configuration of active substance of 1904–1 was assigned to be identical to that of cyclomarin A from the NOESY and ROESY results (Fig 13).

**Table 1:** 1H and 13C NMR data for 1904-1(Cyclomarin A).

	δ_C_			δ_H_		δ_N_ ^1)^
position	this study	ref. 1^2)^	ref. 2^3)^	this study	ref. 2^3)^	this study
1	**170.99**	170.8	171			
2	**52.73**	53.3	52.9	**4.577 (1H, *t*, 3.7 Hz)**	4.58 (*t*, 3)	
3	**68.51**	68.7	68.6	**5.293 (1H, *d*, 3.5 Hz)**	5.31 (*d*, 2.5)	
3-OH				**4.382 (1H, *s*)**		
4	**123.32**	123.4	123.4	**7.322 (1H, *s*)**	7.34 (*s*)	
5	**111.93**	112.1	111.9			
6	**126.9**	127	127.0			
7	**119.06**	119.3	119.2	**7.578 (1H, *d*, 7.9 Hz)**	7.58 (*d*, 8)	
8	**119.82**	119.8	119.8	**7.083 (1H, *t*, 7.4 Hz)**	7.10 (*ddd*, 1,7,7)	
9	**122.11**	122.1	122.1	**7.178 (1H, *dd*, 7.8,1.4 Hz)**	7.17 (*dd*, 8,8)	
10	**113.65**	111.8	113.7	**7.717 (1H, *d*, 8.5 Hz)**	7.74 (*d*, 8.5)	
11	**136.05**	135.9	136.1			
12	**58.12**	58.1	58.1			
13	**57.77**	57.7	57.8	**3.208 (1H, *dd*, 3.8,2.8 Hz)**	3.22 (*dd*, 3,4)	
14a	**45.42**	45.4	45.4	**2.883 (1H, *t*, 4.3 Hz)**	2.90 (*t*, 4)	
14b				**2.745 (1H, *dd*, 4.6,2.7 Hz)**	2.76 (*dd*, 2.5,4.5)	
15	**23.07**	23.1	23.1	**1.555 (3H, *s*)**	1.57 (*s*)	
16	**24.44**	24.4	24.5	**1.649 (3H, *s*)**	1.66 (*s*)	
17	**172.53**	172.4	172.5			
18	**58.08**	58.1	58.1	**4.073 (1H, *t*, 10.0 Hz)**	4.08 (*t*, 10)	
19	**35.53**	35.5	35.5	**1.65 (*m*)**	1.66 (*m*)	
20	**124.76**	124.7	124.8	**4.758 (1H, *d*, 9.9 Hz)**	4.77 (*d*, 10)	
21	**134.47**	134.4	134.4			
22	**25.72**	25.7	25.7	**1.724 (3H, *s*)**	1.26 (*s*)	
23	**18.87**	20.8	18.9	**1.249 (3H, *s*)**	1.73 (*s*)	
24	**18.5**	18.5	18.5	**0.638 (3H, *d*, 6.5 Hz)**	0.64 (*d*, 7.5)	
25	**168.4**	168.6	168.4			
26	**58.62**	58.7	58.6	**4.809 (1H, *dd*, 10.2,3.4 Hz)**	4.83 (*t*, 3.5)	
27a	**38.89**	38.9	38.9	**2.25 (1H, *m*)**	2.25 (4.5,10.5,13.5)	
27b				**1.04 (1H, *m*)**	1.05 (*m*)	
28	**25.04**	25	25.0	**1.40 (1H, *m*)**	1.40 (*m*)	
29	**22.38**	23.4	24.4	**0.820 (3H, *d*, 6.6 Hz)**	0.82 (*d*, 6)	
30	**23.49**	23.5	23.5	**0.870 (3H, *d*, 6.7 Hz)**	0.87 (*d*, 6)	
31	**170.57**	170.8	170.6			
32	**55.24**	55.3	55.2	**4.360 (1H, *t*, 8.6 Hz)**	4.36 (*t*, 8.5)	
33	**30.84**	30.7	30.8	**2.21 (1H, *m*)**	2.20 (*m*)	
34	**19.27**	19.3	19.3	**1.059 (3H, *d*, 6.8 Hz)**	1.06 (*d*, 6.5)	
35	**19.99**	19.9	20.0	**0.935 (3H, *d*, 6.8 Hz)**	0.94 (*d*, 6.5)	
36	**169.59**	169.7	169.6			
37	**55.88**	55.9	55.9	**4.886 (1H, *t*, 5.1 Hz)**	4.90 (*t*, 5)	
38	**79.92**	80.1	80	**5.068 (1H, *d*, 5.4 Hz)**	5.08 (*d*, 5.5)	
39	**135.1**	135.1	135.1			
40 & 44	**127.83**	127∼128	127.8	**7.184 (2H, *dd*, 7.2,1.5 Hz)**	7.24∼7.26 (*m*)	
41 & 43	**128.33**	127∼128	128.3	**7.235 (2H, *m*)**	7.24∼7.26 (*m*)	
42	**128.69**	127∼128	128.7	**7.231 (1H, *t*)**	7.24∼7.26 (*m*)	
45	**57.74**	57.7	57.8	**3.360 (3H, *s*)**	3.37 (*s*)	
46	**171.58**	171.5	171.6			
47	**50.61**	50.6	50.6	**4.869 (1H, *dd*, 10.3,7.3 Hz)**	4.88 (*m*)	
48	**20.82**	21.2	20.8	**1.294 (3H, *d*, 7.3 Hz)**	1.31 (*d*, 7.5)	
49	**168.81**	168.8	168.8			
50	**59.29**	59.2	59.3	**4.793 (1H, *dd*, 10.2,3.3 Hz)**	4.81 (*t*, 8)	
51a	**33.07**	33.1	33.1	**2.30 (1H, *m*)**	2.33 (7.5,11,14.5)	
51b				**0.63 (1H, *m*)**	0.72 (4,6.5,14.5)	
52	**33.2**	32.8	33.2	**1.43 (1H, *m*)**	1.43 (*m*)	
53a	**66.43**	66.3	66.5	**3.15 (1H, *m*)**	3.18 (*m*)	
53b				**3.22 (1H, *m*)**	3.26 (*m*)	
53-OH				**2.498 (1H, *brt*, 5.3 Hz)**		
54	**17.72**	17.6	17.6	**0.728 (3H, *d*, 6.8 Hz)**	0.76 (*d*, 7)	
N-1						**150.8**
NH-2				**6.752 (1H, *d*, 2.3 Hz)**	6.72 (*d*, 4)	**113.9**
NH-3				**8.035 (1H, *d*, 9.5 Hz)**	8.05 (*d*, 10)	**114**
NH-5				**7.931 (1H, *d*, 9.9 Hz)**	7.95 (*d*, 8)	**114.1**
NH-6				**7.117 (1H, *d*, 4.5 Hz)**	7.13 (*d*, 4.5)	**111.7**
NH-7				**8.152 (1H, *d*, 10.3 Hz)**	8.16 (*d*, 10.5)	**114**
NMe-4	**29.53**	29.6		**2.817 (3H, *s*)**	2.83 (*s*)	**121.8**
NMe-8	**29.3**	29.3	29.2	**2.710 (3H, *s*)**	2.73 (*s*)	**120.3**

^1)^ the chemical shifts were calculated against the external standard for 15N; N-Urea with 75.0 ppm in DMSO-d6 soln.

^2)^ the values cited from the ref.1 (measured in the same condition except that measured by 500MHz NMR)

^3)^ the values cited from the ref.2

ref.1, Matthew K. Renner et al., J. Am. Chem. Soc., 1999,121,11273–11276

ref.2, Andrew W. Schultz et al., J. Am. Chem. Soc., 2008,130,4507–4516.

### Genome analysis of the mutant BCG strain resistant to the active substance of 1904–1

**Fig 14:**
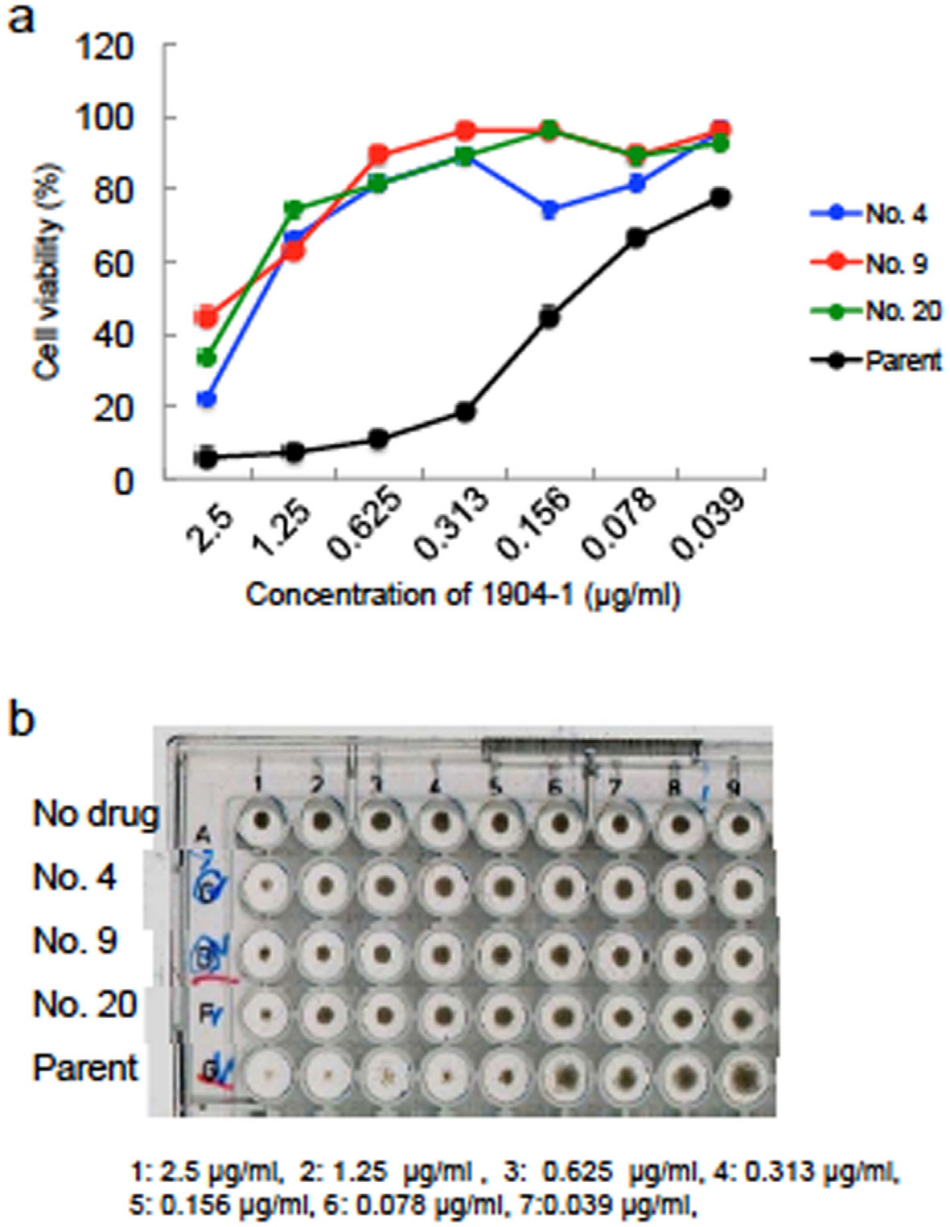
Susceptibility testing of resistant BCG mutants to the active substance of 1904–1. Three BCG strains resistant to the active substance of the 1904–1 extract were obtained by selection on agar containing the substance and are designated as 4, 9, and 20. The upper graph (a) and lower picture (b) show the results of susceptibility tests of these mutants to the active substance in the media according to luciferase activity (a) and macroscopic examination (b), respectively. Bacteria were cultured in the media containing the indicated concentrations of the substance for 4 days for the luciferase assay and 9 days for macroscopic examination. Parent, parental rBCG-MDP1-luc. No. 4, 9, and 20, resistant strains to active substance of 1904–1. The representative data of two independent experiments are presented.

**Fig 15:**
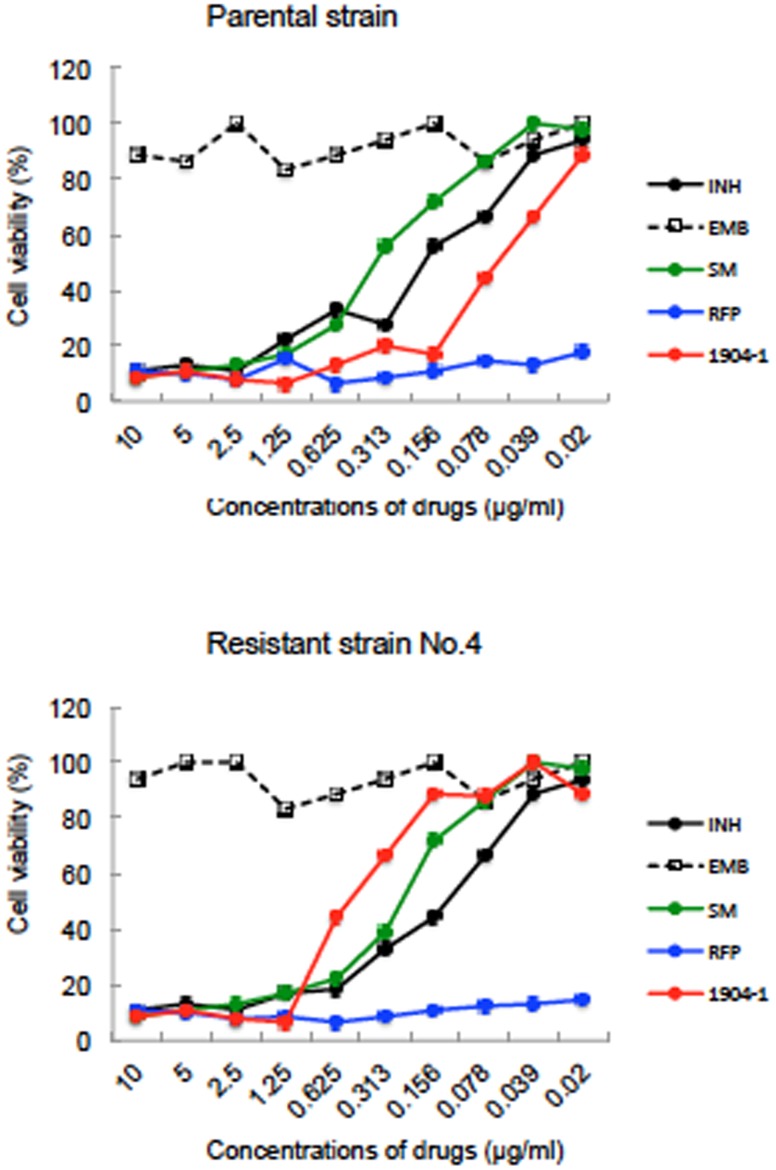
Susceptibility test of resistant strain, No. 4 against conventional TB drugs. Parental rBCG-MDP1-luc and its resistant derivative; No. 4 were cultured in the media containing isoniazid (INH), ethambutol (EMB), streptomycin (SM), rifampicin (RFP), active substance of 1904–1. Four days after culture, luciferase activity was measured. The data were normalized against medium alone. The representative data of two independent experiments are presented.

It was reported that cyclomarin A targets the caseinolytic protein C1 (ClpC1) of Mtb [27, 28]. To confirm this mode of action for the active substance of 1904–1, we obtained 3 rBCG-MDP1-luc strains resistant to the active substance. The frequency of the emergence of resistant colonies was around 1/10^8^ CFU, which is equivalent to that of RFP. The MIC values of all 3 obtained isolates were 1.25–2.5 μg/ml, while that of the parental rBCG-MDP1-luc was 0.156 μg/ml by macroscopic examination (Fig 14a and 14b). The susceptibility of these resistant strains to other anti-TB drugs was similar to those of the parental strain (Fig 15).

We also extracted genomic DNA from the 3 resistant isolates and their parental strain and then performed a comparative analysis based on whole genome sequencing. Draft *de novo* genome assembly and the comparison of the parental and resistant strains showed that all 3 strains contained mutations in *clpc* (BCG3661c), an equivalent gene to *clpc1* [23, 29], which is essential and in two non-essential genes, *prcA* (proteasome alpha subunit) [30] and *ppsE* (phenol synthesis type-1 polyketide synthase) (Table 2) [31]. This unambiguously suggested that the target of the active substance of 1904–1 is *clpc1*. Moreover, we highlighted how a drug screening system using a rBCG expressing luciferase is useful for discovering TB drug candidates in a timely fashion.

**Table 2:** Genomic analysis of the 3 resistant isolates.

Start Position in Ref	Reference Bases	Variation Bases	No. of strains	Total depth			gene	gene type	Product	coding_var	ref_AA	mut_AA	ref_codon	mut_codon
				BCG4	BCG9	BCG20								
893072	-	C	1		9		JTY_0819	misc_feature	non functional due to frameshift					
960701	C	-	1	8						intergenic				
1093832	A	C	1	4			PE_PGRS 16	CDS	PE-PGRS family protein	Nonsynonymous	Q	H	caa	cac
1093833	T	G	1	4			PE_PGRS 16	CDS	PE-PGRS family protein	Nonsynonymous	C	G	tgc	ggc
1094037	A	C	1			7	PE_PGRS 16	CDS	PE-PGRS family protein	Nonsynonymous	K	N	aaa	aac
1094043	T	C	1			8	PE_PGRS 16	CDS	PE-PGRS family protein	silent				
1191370	A	C	1	3			PE_PGRS 19	CDS	PE-PGRS family protein					
2758356	-	A	1		8		PE_PGRS43b		PE-PGRS family protein					
893038	-	A	2	6	9		JTY_0819	misc_feature	non functional due to frameshift					
1093871	A	C	2	4		4	PE_PGRS 16	CDS	PE-PGRS family protein	silent				
1093878	A	G	2	4		4	PE_PGRS 16	CDS	PE-PGRS family protein	Nonsynonymous	R	G	aga	gga
1093880	A	C	2	4		4				Nonsynonymous	R	S	aga	agc
1093883	A	G	2	4		4	PE_PGRS 16	CDS	PE-PGRS family protein	silent				
3851427	C	T	2		23	40	JTY_3516	CDS	hypothetical protein	silent				
**2342188**	**C**	**T**	**3**	**47**	**37**	**52**	**prcA**	**CDS**	**proteasome subunit alpha**	**Nonsynonymous**	**E**	**K**	**gag**	**aag**
**3215081**	**A**	**C**	**3**	**46**	**38**	**46**	**ppsE**	**CDS**	**phenolpthiocerol synthesis type-I polyketide synthase**	**Nonsynonymous**	**S**	**R**	**agc**	**cgc**
**4016743**	**A**	**C**	**3**	**37**	**34**	**40**	**clpC**	**CDS**	**putative ATP-dependent Clp protease ATP-binding subunit**	**Nonsynonymous**	**F**	**V**	**ttt**	**gtt**

Gene mutations common to 3 resistant strains are indicated by boldface.

## Discussion

TB is a serious life-threatening disease resulting in approximately 9 million cases and 1.5 million deaths annually. A major problem of TB treatment is the extraordinary long duration of the therapy. To shorten the duration of TB chemotherapy and effectively treat emergent drug resistant TB strains including MDR-TB and XDR-TB, the development of new drugs with different modes of action is urgently required.

A luciferase reporter system using Mtb-infectious phages was expected to be useful for the rapid screening of drug resistance Mtb in a clinical setting. However, the unstable infectious rate of the phage in the clinical strains has impeded its adaptation to a clinical setting. In contrast, the laboratory-based construction of rBCG-MDP1-luc stably expresses luciferase. Recently Singh et al showed usefulness of rBCG expressing luciferase [32] using promoters of HSP60 and *kas* operon which is responding to the inhibition of cell wall synthesis in mycobacteria [33]. MDP1 is an essential protein[22, 23] and is constitutively expressed even in the dormant state [34]. Utilizing the promoter of MDP1 and the stable multicopy plasmid “pSO246” may contribute to the stable detection of anti-BCG efficacy [35]. The usage of rBCG-MDP1-luc for screening of drugs against the dormant Mtb is still under investigation.

As expected, the luciferase activity of rBCG-MDP1-luc correlated well with the number of viable bacteria and can detect over 10^3^ CFU of viable BCG. We found that luciferase-based detection of anti-mycobacterial efficacy is sensitive to DNA, RNA, and protein synthesis inhibitors but relatively insensitive to the inhibitors of cell wall synthesis. This is because of the continuous expression of luciferase and the retarded degradation of cellular ATP after the cessation of cell wall synthesis.

We conducted a drug screening campaign employing 10,080 individual extracts from *Actinomyces* and *Streptomyces* using a combination of rBCG-MDP1-luc and Mtb strains. We found 137 effective extracts against rBCG-MDP1-luc at dilutions up to 1:1600, however among them, only 41 extracts were effective up to a 1:200 dilution against laboratory strain, H37Rv and at least one of 2 XDR-Mtb strains. This discrepancy can be explained by the fact that some extracts originally included anti-TB drugs identified in actinomycetes such as KM, SM, and amikacin. Secondly, the cell wall permeability is low in XDR-Mtb strains compared with that of rBCG and drug susceptible Mtb, thus, effective substances in the extracts may not reach their targets in XDR-Mtb strains. This data emphasized the importance of combining the efficacy tests against both laboratory and clinical strains for TB drug screening.

Among the 10,080 tested extracts, 1904–1 showed strong bactericidal activity and the lowest toxicity toward the eukaryotic cells. Thus, we conducted further characterization and determination of the effective substance of the 1904–1 extract. We developed a large-scale purification system and succeeded in the isolation of 22 mg of active substance from 100 ml culture extracts. The MIC values of the purified active substance were 0.5, 2.0, and 7.5 μg/ml against Mtb H37Rv, XDR-Mtb5, and XDR-Mtb44, respectively, indicating the potential of the active substance from the 1904–1 extract as a possible TB drug. MS and NMR analyses showed that the active substance of 1904–1 was cyclomarin A, recently identified as an inhibitor of *clpc1*, which is an essential component of an ATP-dependent proteinase.

Despite the large scale of the screening and the limitation of the biological resources, we and another group recently identified cyclomarin A as a potential TB drug [28]. A combination of structural biology and *in silico* analysis of inhibitors of essential enzymes will help to identify effective compounds with different modes of action from those of the currently available drugs. Ultimately, our constructed rBCG expressing luciferase may be used as an effective tool to advance drug development.

## Supporting Information

S1 Fig Isolation of the active fraction of the 1904–1 extract by HPLC with methanol. The BtOH layer of the 1904–1 extract was analyzed by HPLC with methanol as a mobile phase in preparative trial. (a) UV spectroscopic data at 254 nm. (b) The retention times of the eluents. The luciferase activity of rBCG-MDP1-luc in each well was monitored 4 days after culture (c) and the macroscopic growth was checked 9 days after culture (d). The retention time of the active substances are circled in (a) and marked by red in (b). The representative data of two independent experiments are presented.
(TIF)

S2 Fig Large-scale isolation of the active fraction of the 1904–1 extract. The active fraction was obtained with methanol as a mobile phase using large-scale HPLC. (a) The active fraction (peak 2) was isolated. The ESI-mass spectrum of the active fraction from positive and negative ion mode acquisition was shown in (b) and (c), respectively.
(TIF)

S3 Fig Efficacy of active substance of 1904 to H37Rv in the stationary growth phase. Mtb H37Rv was grown until stationary growth phase (OD_600_ = 0.87) and 100 μl of culture was transferred into a-96-well round bottom plate with or without 1μl of active substance at various concentrations. Three days after inoculation, the numbers of viable bacterial cells were determined by using BacTiter-Glo Microbial Cell Viability Assay kit (Promega, WI), which based on quantification of ATP of viable bacterial cells. Data were normalized against bacterial suspension alone.
(TIF)

S4 Fig Cytotoxity of 1904–1 to THP1 cells. The THP1 cells (1x10^5^ cells/ml, 100 μl/well) were cultured with active substance of 1904-1dissolved in DMSO: cremophor: saline (1:1:8; Cremo) or DMSO: 0.1% BSA in PBS (1:9; BSA) or DMSO: saline (1:9; saline). Three days after cultivation, the cell viability was evaluated using the cell proliferation reagent WST-1. Increases of respiration were monitored at 2 h after adding the redox-sensitive growth indicator. The representative data of two independent experiments are presented as means ± SD.
(TIF)

S1 Table MIC of XDR-Mtb. Susceptibility of XDR-Mtb5 and XDR-Mtb44 to anti-TB drugs was indicated.
(XLSX)

S2 Table Mutations of drug resistant-related genes in XDR-Mtb strains. Genetic mutations of XDR-Mtb5 and XDR-Mtb44 were indicated.
(XLSX)

S3 Table Effective extracts of actinomycetes. The 41 effective extracts against rBCG-MDP1-luc, Mtb H37Rv, XDR-Mtb5, and XDR-Mtb44 were listed.
(XLSX)

## References

[1] WHO. Available from: http://apps.who.int/iris/bitstream/10665/137094/1/9789241564809_eng.pdf.

[2] Raviglione MC, Smith IM. XDR tuberculosis—implications for global public health. N Engl J Med. 2007;356(7):656–9. Epub 2007/02/16. 356/7/656 [pii] 10.1056/NEJMp068273 . 17301295

[3] Van Rie A, Enarson D. XDR tuberculosis: an indicator of public-health negligence. Lancet. 2006;368(9547):1554–6. Epub 2006/11/07. S0140-6736(06)69575-5 [pii] 10.1016/S0140-6736(06)69575-5 . 17084741

[4] Gler MT, Skripconoka V, Sanchez-Garavito E, Xiao H, Cabrera-Rivero JL, Vargas-Vasquez DE, et al Delamanid for multidrug-resistant pulmonary tuberculosis. N Engl J Med. 2012;366(23):2151–60. 10.1056/NEJMoa1112433 . 22670901

[5] Cox E, Laessig K. FDA approval of bedaquiline—the benefit-risk balance for drug-resistant tuberculosis. N Engl J Med. 2014;371(8):689–91. 10.1056/NEJMp1314385 . 25140952

[6] Burki T. Improving the health of the tuberculosis drug pipeline. Lancet Infect Dis. 2014;14(2):102–3. . 24605379 10.1016/s1473-3099(14)70006-4

[7] Jacobs WR Jr., Tuckman M, Bloom BR. Introduction of foreign DNA into mycobacteria using a shuttle phasmid. Nature. 1987;327(6122):532–5. Epub 1987/06/11. 10.1038/327532a0 . 3473289

[8] Grode L, Seiler P, Baumann S, Hess J, Brinkmann V, Nasser Eddine A, et al Increased vaccine efficacy against tuberculosis of recombinant Mycobacterium bovis bacille Calmette-Guerin mutants that secrete listeriolysin. J Clin Invest. 2005;115(9):2472–9. Epub 2005/08/20. 10.1172/JCI24617 16110326 PMC1187936

[9] Horwitz MA, Harth G, Dillon BJ, Maslesa-Galic S. Recombinant bacillus calmette-guerin (BCG) vaccines expressing the Mycobacterium tuberculosis 30-kDa major secretory protein induce greater protective immunity against tuberculosis than conventional BCG vaccines in a highly susceptible animal model. Proc Natl Acad Sci U S A. 2000;97(25):13853–8. Epub 2000/11/30. 10.1073/pnas.250480397 [pii]. 11095745 PMC17665

[10] Aldovini A, Young RA. Humoral and cell-mediated immune responses to live recombinant BCG-HIV vaccines. Nature. 1991;351(6326):479–82. Epub 1991/06/06. 10.1038/351479a0 . 2046750

[11] Langermann S, Palaszynski S, Sadziene A, Stover CK, Koenig S. Systemic and mucosal immunity induced by BCG vector expressing outer-surface protein A of Borrelia burgdorferi. Nature. 1994;372(6506):552–5. Epub 1994/12/08. 10.1038/372552a0 . 7990928

[12] Langermann S, Palaszynski SR, Burlein JE, Koenig S, Hanson MS, Briles DE, et al Protective humoral response against pneumococcal infection in mice elicited by recombinant bacille Calmette-Guerin vaccines expressing pneumococcal surface protein A. J Exp Med. 1994;180(6):2277–86. Epub 1994/12/01. 7964500 10.1084/jem.180.6.2277 PMC2191795

[13] Matsumoto S, Yukitake H, Kanbara H, Yamada T. Recombinant Mycobacterium bovis bacillus Calmette-Guerin secreting merozoite surface protein 1 (MSP1) induces protection against rodent malaria parasite infection depending on MSP1-stimulated interferon gamma and parasite-specific antibodies. J Exp Med. 1998;188(5):845–54. Epub 1998/09/09. 9730886 10.1084/jem.188.5.845 PMC2213399

[14] Stover CK, de, la, Cruz Vf, Fuerst TR, et al New use of BCG for recombinant vaccines. Nature. 1991;351(6326):456–60. 1904554 10.1038/351456a0

[15] Yamada H, Matsumoto S, Matsumoto T, Yamada T, Yamashita U. Murine IL-2 secreting recombinant Bacillus Calmette-Guerin augments macrophage-mediated cytotoxicity against murine bladder cancer MBT-2. J Urol. 2000;164(2):526–31. Epub 2000/07/14. S0022-5347(05)67417-4 [pii]. . 10893638

[16] de Wet JR, Wood KV, Helinski DR, DeLuca M. Cloning of firefly luciferase cDNA and the expression of active luciferase in *Escherichia coli* . Proc Natl Acad Sci USA. 1985;82(23):7870–3. . 3906652 10.1073/pnas.82.23.7870 PMC390871

[17] Alcaide F, Gali N, Dominguez J, Berlanga P, Blanco S, Orus P, et al Usefulness of a new mycobacteriophage-based technique for rapid diagnosis of pulmonary tuberculosis. J Clin Microbiol. 2003;41(7):2867–71. Epub 2003/07/05. 12843014 10.1128/JCM.41.7.2867-2871.2003 PMC165270

[18] Jacobs WR Jr., Barletta RG, Udani R, Chan J, Kalkut G, Sosne G, et al Rapid assessment of drug susceptibilities of Mycobacterium tuberculosis by means of luciferase reporter phages. Science. 1993;260(5109):819–22. Epub 1993/05/07. . 8484123 10.1126/science.8484123

[19] Katsube T, Matsumoto S, Takatsuka M, Okuyama M, Ozeki Y, Naito M, et al Control of cell wall assembly by a histone-like protein in Mycobacteria. Journal of bacteriology. 2007;189(22):8241–9. . 17873049 10.1128/JB.00550-07 PMC2168677

[20] Matsumoto S, Tamaki M, Yukitake H, Matsuo T, Naito M, Teraoka H, et al A stable *Escherichia coli*-mycobacteria shuttle vector 'pSO246' in *Mycobacterium bovis* BCG. FEMS Microbiol Lett. 1996;135(2–3):237–43. 8595863 10.1016/0378-1097(95)00457-2

[21] Matsumoto S, Yukitake H, Furugen M, Matsuo T, Mineta T, Yamada T. Identification of a novel DNA-binding protein from *Mycobacterium bovis* bacillus Calmette-Guerin. Microbiol Immunol. 1999;43(11):1027–36. . 10609612 10.1111/j.1348-0421.1999.tb01232.x

[22] Rao A, Ram G, Saini AK, Vohra R, Kumar K, Singh Y, et al Synthesis and selection of de novo proteins that bind and impede cellular functions of an essential mycobacterial protein. Appl Environ Microbiol. 2007;73(4):1320–31. AEM.02461-06 [pii] 10.1128/AEM.02461-06 . 17189438 PMC1828669

[23] Sassetti CM, Boyd DH, Rubin EJ. Genes required for mycobacterial growth defined by high density mutagenesis. Mol Microbiol. 2003;48(1):77–84. . 12657046 10.1046/j.1365-2958.2003.03425.x

[24] Staneck JL, Roberts GD. Simplified approach to identification of aerobic actinomycetes by thin-layer chromatography. Appl Microbiol. 1974;28(2):226–31. 4605116 10.1128/am.28.2.226-231.1974 PMC186691

[25] Schultz AW, Oh DC, Carney JR, Williamson RT, Udwary DW, Jensen PR, et al Biosynthesis and structures of cyclomarins and cyclomarazines, prenylated cyclic peptides of marine actinobacterial origin. J Am Chem Soc. 2008;130(13):4507–16. 10.1021/ja711188x. Epub 2008 Mar 11. 18331040

[26] Renner MK, Shen Y-C, Cheng X-C, Jensen PR, Frankmoelle W, Kauffman CA, et al Cyclomarins A-C, New Antiinflammatory Cyclic Peptides Produced by a Marine Bacterium (*Streptomyces* sp.). *J Am Chem Soc*. 1999;121:11273–6.

[27] Ollinger J, O'Malley T, Kesicki EA, Odingo J, Parish T. Validation of the essential ClpP protease in Mycobacterium tuberculosis as a novel drug target. J Bacteriol. 194(3):663–8. Epub 2011/11/30. JB.06142-11 [pii] 10.1128/JB.06142-11 22123255 PMC3264079

[28] Schmitt EK, Riwanto M, Sambandamurthy V, Roggo S, Miault C, Zwingelstein C, et al The natural product cyclomarin kills Mycobacterium tuberculosis by targeting the ClpC1 subunit of the caseinolytic protease. Angew Chem Int Ed Engl. 50(26):5889–91. Epub 2011/05/13. 10.1002/anie.201101740 . 21563281

[29] Ollinger J, O'Malley T, Kesicki EA, Odingo J, Parish T. Validation of the essential ClpP protease in *Mycobacterium tuberculosis* as a novel drug target. Journal of bacteriology. 2012;194(3):663–8. Epub 2011/11/30. JB.06142-11 [pii] 10.1128/JB.06142-11 22123255 PMC3264079

[30] Gandotra S, Lebron MB, Ehrt S. The *Mycobacterium tuberculosis* proteasome active site threonine is essential for persistence yet dispensable for replication and resistance to nitric oxide. PLoS Pathog. 2010;6(8):e1001040 Epub 2010/08/17. 10.1371/journal.ppat.1001040 20711362 PMC2920845

[31] Lamichhane G, Zignol M, Blades NJ, Geiman DE, Dougherty A, Grosset J, et al A postgenomic method for predicting essential genes at subsaturation levels of mutagenesis: application to Mycobacterium tuberculosis. Proc Natl Acad Sci U S A. 2003;100(12):7213–8. Epub 2003/05/31. 10.1073/pnas.1231432100 [pii]. 12775759 PMC165855

[32] Singh V, Biswas RK, Singh BN. Double recombinant *Mycobacterium bovis* BCG strain for screening of primary and rationale-based antimycobacterial compounds. Antimicrob Agents Chemother. 2014;58(3):1389–96. 10.1128/AAC.01301-13 24342633 PMC3957833

[33] Mdluli K, Slayden RA, Zhu Y, Ramaswamy S, Pan X, Mead D, et al Inhibition of a *Mycobacterium tuberculosis* beta-ketoacyl ACP synthase by isoniazid. Science. 1998;280(5369):1607–10. . 9616124 10.1126/science.280.5369.1607

[34] Lewin A, Baus D, Kamal E, Bon F, Kunisch R, Maurischat S, et al The mycobacterial DNA-binding protein 1 (MDP1) from *Mycobacterium bovis* BCG influences various growth characteristics. BMC Microbiol. 2008;8(1):91 . 18544159 10.1186/1471-2180-8-91 PMC2453136

[35] Matsumoto S, Tamaki M, Yukitake H, Matsuo T, Naito M, Teraoka H, et al A stable Escherichia coli-mycobacteria shuttle vector 'pSO246' in Mycobacterium bovis BCG. FEMS Microbiol Lett. 1996;135(2–3):237–43. Epub 1996/01/15. . 8595863 10.1016/0378-1097(95)00457-2

