# Supplementary figures and images for: A New Screen for Tuberculosis Drug Candidates Utilizing a Luciferase-Expressing Recombinant Mycobacterium bovis Bacillus Calmette-Guéren
Source: PLoS One. 2015 Nov 16;10(11):e0141658. doi: 10.1371/journal.pone.0141658 (PMC4646695; doi:10.1371/journal.pone.0141658)

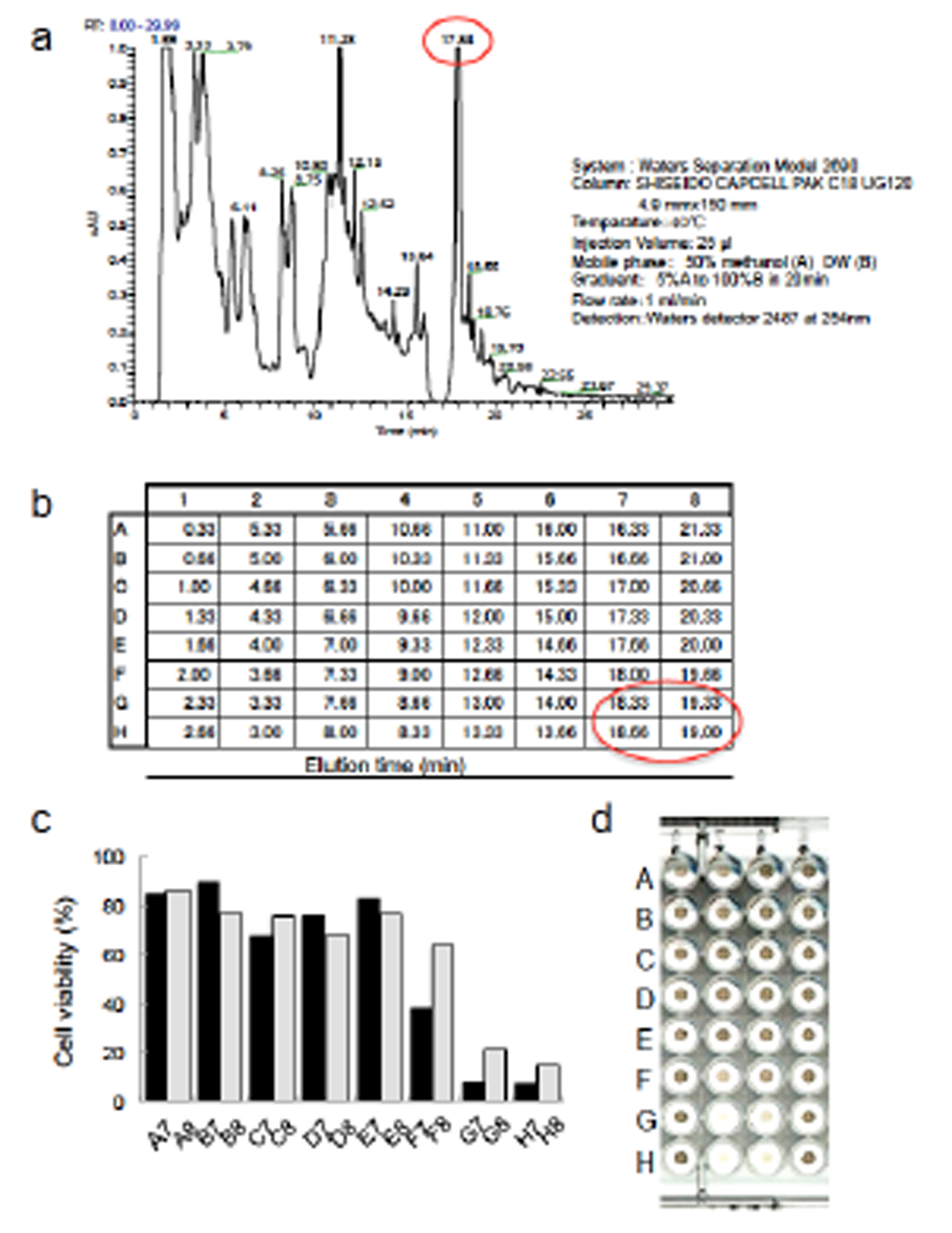

Supplement: S1 Fig — The BtOH layer of the 1904–1 extract was analyzed by HPLC with methanol as a mobile phase in preparative trial. (a) UV spectroscopic data at 254 nm. (b) The retention times of the eluents. The luciferase activity of rBCG-MDP1-luc in each well was monitored 4 days after culture (c) and the macroscopic growth was checked 9 days after culture (d). The retention time of the active substances are circled in (a) and marked by red in (b). The representative data of two independent experiments are presented. (TIF) [file pone.0141658.s001.tif]

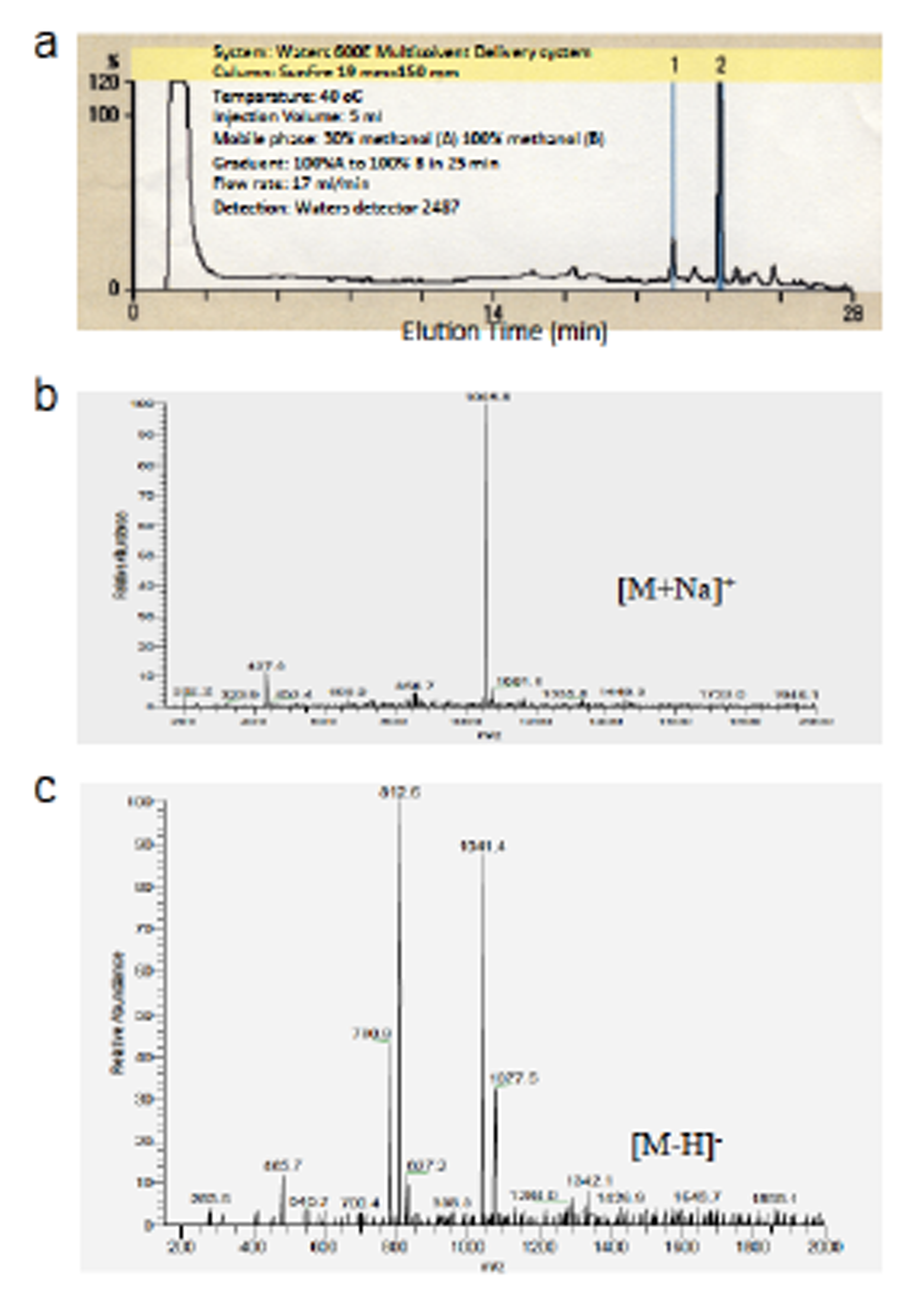

Supplement: S2 Fig — The active fraction was obtained with methanol as a mobile phase using large-scale HPLC. (a) The active fraction (peak 2) was isolated. The ESI-mass spectrum of the active fraction from positive and negative ion mode acquisition was shown in (b) and (c), respectively. (TIF) [file pone.0141658.s002.tif]

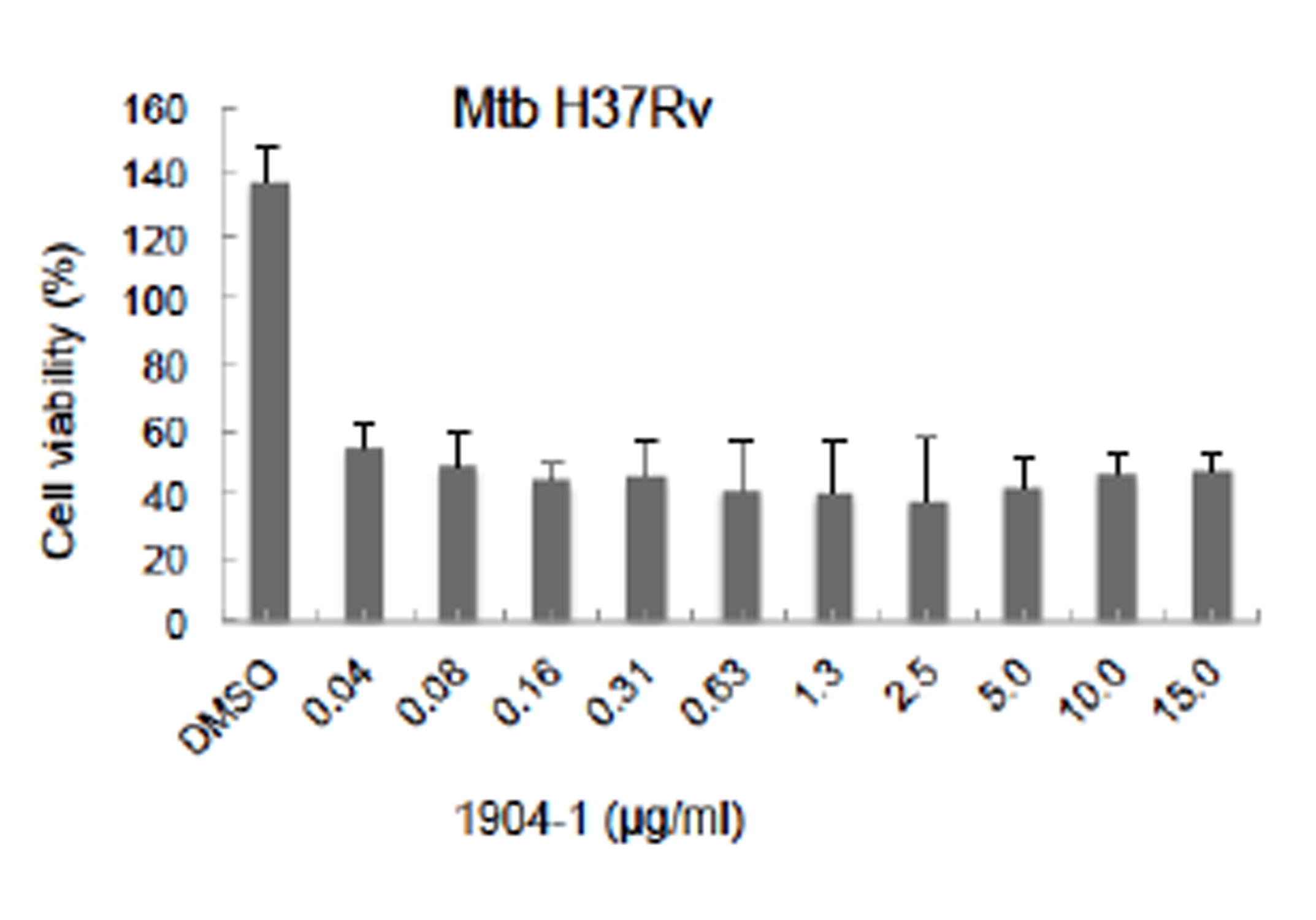

Supplement: S3 Fig — Mtb H37Rv was grown until stationary growth phase (OD600 = 0.87) and 100 μl of culture was transferred into a-96-well round bottom plate with or without 1μl of active substance at various concentrations. Three days after inoculation, the numbers of viable bacterial cells were determined by using BacTiter-Glo Microbial Cell Viability Assay kit (Promega, WI), which based on quantification of ATP of viable bacterial cells. Data were normalized against bacterial suspension alone. (TIF) [file pone.0141658.s003.tif]

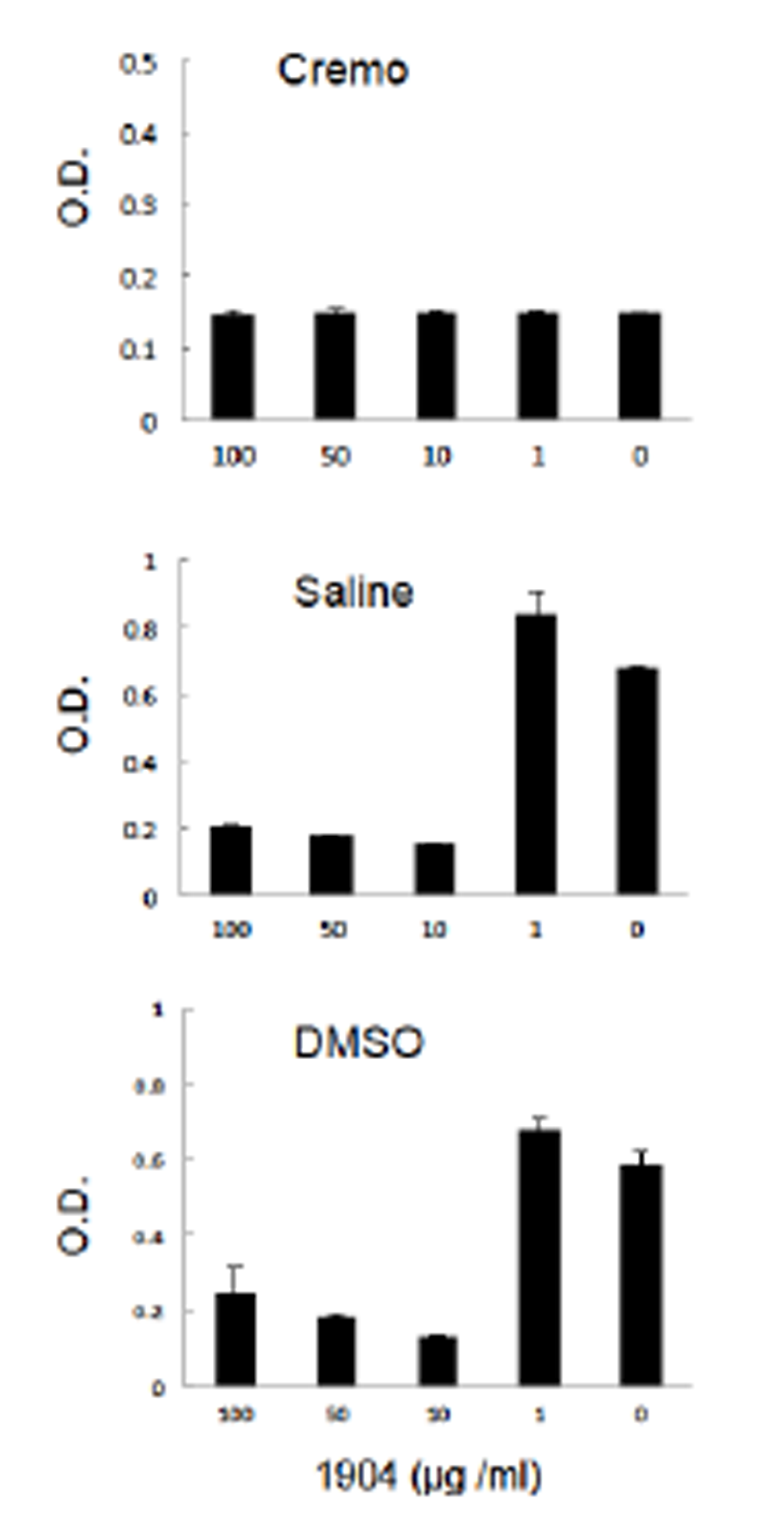

Supplement: S4 Fig — The THP1 cells (1x10^5^ cells/ml, 100 μl/well) were cultured with active substance of 1904-1dissolved in DMSO: cremophor: saline (1:1:8; Cremo) or DMSO: 0.1% BSA in PBS (1:9; BSA) or DMSO: saline (1:9; saline). Three days after cultivation, the cell viability was evaluated using the cell proliferation reagent WST-1. Increases of respiration were monitored at 2 h after adding the redox-sensitive growth indicator. The representative data of two independent experiments are presented as means ± SD. (TIF) [file pone.0141658.s004.tif]
